# CircSCAP interacts with SF3A3 to inhibit the malignance of non-small cell lung cancer by activating p53 signaling

**DOI:** 10.1186/s13046-022-02299-0

**Published:** 2022-04-01

**Authors:** Dongni Chen, Hongli Zhou, Zhuochen Cai, Kaican Cai, Ji Liu, Weidong Wang, Huikai Miao, Hongmu Li, Rongzhen Li, Xiaodong Li, Youfang Chen, Hui-Yun Wang, Zhesheng Wen

**Affiliations:** 1 Department of Thoracic Oncology, State Key Laboratory of Oncology in South China, Collaborative Innovation Center for Cancer Medicine, Sun Yat-sen University Cancer Center, Guangzhou, 510060 China; 2 Department of Thoracic Surgery, Nanfang Hospital, Southern Medical University, Guangzhou, 510089 China; 3 Key Laboratory of Tropical Disease Control, Zhongshan School of Medicine, Sun Yat-Sen University, Guangzhou, China; 4 State Key Laboratory of Oncology in South China, Collaborative Innovation Center for Cancer Medicine, Sun Yat-Sen University Cancer Center, Guangzhou, 510060 China

**Keywords:** Non-small cell lung cancer, CircSCAP, SF3A3, p53, Prognosis

## Abstract

**Background:**

Circular RNA (circRNA) has been recently identified as a critical regulator during carcinogenesis. However, the biological function and potential underlying mechanisms of circRNAs in lung cancer remain to be further elucidated.

**Methods:**

Here, we first evaluated the differentially expressed circRNAs between tumor and the matched adjacent nontumor tissues (3 pairs) of lung cancer patients via circRNA microarray. The expression of top five dysregulated circRNAs were tested in lung cancer cell lines and the circSCAP with concordant alteration in microarray data and cell lines was selected for further investigation. Then we validated the expression level of circSCAP in tumor and corresponding adjacent tissues (161 pairs) from a lung cancer cohort by RT-PCR analysis followed by correlation and prognosis analysis between circSCAP and clinical characteristics. Non-small cell lung cancer (NSCLC) accounts for the majority of lung cancer diagnosis (about 80% in the cohort used in this study). Therefore, we focused the role of circSCAP in NSCLC in the present study. In vitro and in vivo assays were performed to study the biological function of circSCAP in NSCLC. Biotin-labeled RNA pulldown and RNA immunoprecipitation (RIP) assays were carried out to identify the proteins directly interacting with circSCAP. The molecular mechanism of circSCAP-driven tumor suppression was demonstrated by immunoblotting, immunoprecipitation and luciferase reporter assays. In vitro and in vivo rescue experiments were conducted to verify the role of the circSCAP/SF3A3/p53 signaling axis in NSCLC.

**Results:**

We screened the expression profiles of human circRNAs in lung cancer tissues and found that hsa_circ_0065214 (termed as circSCAP) was significantly decreased. Kaplan–Meier analysis showed that patients with low level of circSCAP had a significantly poor prognosis. Gain- and loss-of-function experiments suggested that circSCAP played an important role in NSCLC cell proliferation, cell migration and apoptosis. Mechanistically, circSCAP directly binds to the SF3A3 protein, facilitating the reduction of SF3A3 by promoting its ubiquitin–proteasome-mediated degradation, which enhances the expression of MDM4-S to finally activate its downstream p53 signaling.

**Conclusion:**

These findings illustrate a novel circSCAP/SF3A3/p53 signaling axis involved in suppressing the malignance of NSCLC and provide a promising target for NSCLC prognosis prediction and treatment.

**Supplementary Information:**

The online version contains supplementary material available at 10.1186/s13046-022-02299-0.

## Background

Lung cancer accounts for approximately 10% of all new tumor cases and have the highest mortality rate (approximately 22%) among all cancers [1]. Due to advances in the treatment of lung cancer such as chemotherapy, epidermal growth factor receptor tyrosine kinase inhibitors (EGFR-TKIs) and immunotherapy [2–4], the prognosis and survival have been significantly improved. Nevertheless, a large proportion of lung cancer patients suffered resistance to therapy and poor prognosis [5]. Effective prognosis evaluation would be helpful for treatment decision making and novel targeting therapy is much needed, while there is no such robust biomarkers or targets [4]. Lung cancer mainly consists non-small cell lung cancer (NSCLC) and small cell lung cancer (SCLC), among which, NSCLC accounts for more than 80% of lung cancer diagnosis. Therefore, it is urgent to further demonstrate the pathogenesis and mechanism of lung cancer (especially NSCLC) and identify novel targets to improve treatment outcomes and prognosis evaluation.

Circular RNAs (circRNAs), a special group of endogenous RNAs with covalently closed loop structures, are involved in multiple biological processes [6]. Previous studies document that circRNAs function by acting as microRNA (miRNA) sponges [7], protein binding scaffolds [8], or transcriptional regulators [9]. In particular, circRNAs have shown great potential as targets for prognosis prediction and therapy in NSCLC [10, 11]. For example, the circSATB2 was reported to participate in the progression of NSCLC and could be a potential biomarker for prognosis prediction [10]. However, the functions and mechanisms of the majority of circRNAs in NSCLC remain largely unexplored.

p53 signaling plays an important role in cancer [12, 13]. Mutation and wild-type p53 might exert the opposite functions [12–14]. p53 mutations, mainly missense mutations, were observed in many human cancers and reported to be associated with the development of cancers and therapeutic failure [14]. Wild-type p53 has been recognized to prompt apoptosis, cell cycle arrest, and senescence by binding to DNA to control the expression of target genes in cancer cells [12, 13]. Accumulating studies indicate that posttranslational modifications play important roles in regulating wild-type p53 stability and activity [13]. The phosphorylation of the C-terminal Ser392 functions in the tumor suppressive responses of p53 to ultraviolet (UV) light and inhibits skin cancer [15]. N-terminal phosphorylation of human p53 at Ser15 and Ser20 results in an increase in p53 expression and helps p53 escape MDM2-mediated ubiquitination and degradation [16]. In addition, human p53 can also be phosphorylated at Ser46 after DNA damage [17]. Previous studies have reported that Ser46 phosphorylation of p53 might be critical to suppress the cell cycle of tumor cells [17, 18]. Taken together, these data indicate that wild-type p53 plays a vital role in the progression of malignancy and that activating this signaling is a critical mechanism of tumor suppression.

In the present study, we identified a novel circular RNA named circSCAP, consisting of exons 3, 4, and 5 of its host gene SCAP, as a tumor suppressor in NSCLC. We discovered that circSCAP was significantly downregulated in lung cancer tissues, and inhibited the proliferation, migration but promoted the apoptosis of NSCLC cells in vitro and in vivo. Mechanistically, circSCAP directly binds to splicing factor 3a subunit 3 (SF3A3) and promotes its ubiquitination, leading to degradation of the SF3A3 protein, which enhances the expression of MDM4-S and activating downstream p53 signaling. Altogether, our data illustrate a circSCAP/SF3A3/p53 signaling axis involved in the progression of NSCLC and identify circSCAP as a promising target for prognosis prediction and targeting therapy.

## Methods

### Patient samples

Samples of 173 lung cancer patients were collected from Sun Yat-sen University Cancer Center (SYSUCC), Guangzhou, China between January 2013 and December 2014. Among them, 161 paired lung cancer and adjacent normal tissues were used for the verification of expression level of circSCAP. All patients were free from neoadjuvant therapy before surgery. Clinic pathological features included age, gender, histological type, T stage, N stage, TNM stage, vessel invasion and adjuvant treatment were recorded. The median follow-up time was 32.5 months (range: 1–80 months). Our study was approved by the Ethics Committee of SYSUCC, the reference number is GZR 2018–120. Informed consent was obtained from each patient.

### Cell culture

Human NSCLC cell lines H1650 (wild-type *p53*), A549 (wild-type *p53*), H1299 (*p53* null), PC9 (inactivating *p53* mutation) and HCC827 (inactivating *p53* mutation) and the normal human lung epithelial cell line 16HBE were purchased from the American Type Culture Collection (ATCC, Manassas VA, USA), authenticated by short tandem repeat (STR) profiling, and resuscitated within 6 months. The cell lines were cultured in RPMI 1640 (Gibco), containing 10% FBS (Gibco) and 1% penicillin–streptomycin at 37℃ in a humidified air with 5% CO2. Antibiotics were removed during validation and characterization experiments.

### Cell transfection

Lentivirus-circSCAP and lentivirus-vector were purchased from GenePharma (Suzhou, China). Lentiviruses were ultracentrifuged, concentrated, and validated. A specific small interfering RNA for hsa_circ_0065214 was designed to target the covalent closed junction. All si RNAs, si NC (negative control), microRNA (miRNA) mimics and inhibitors were also purchased from GenePharma (Suzhou, China) (Table S1). PcDNA3.1( +)-SF3A3 (Umine Biotechnology Co., LTD., Guangzhou, China), pmirGLO- hsa_circ_0065214 (GeneCopoeia, USA), and pp53-TA-luc (Beyotime Biotechnology, Shanghai, China) and pySEAP (Kelei biological Technology Co., LTD., China) were transformed into DH5α competent cells, coated plates, and selected monoclonal colony to multiply. All of them were transfected using Lipofectamine 3000 (Invitrogen, Carlsbad, CA, USA).

### RNA extraction, quantitative and real-time PCR (qRT-PCR) detection

The total RNA was extracted from collected cells using TRIzol. RNA concentration was measured using NanoDrop 2000 Spectrophotometer (Thermo Scientific, Wilmington, DE, USA). Then, 2 µg of total RNA was reverse transcribed in a final volume of 20 µL with the Prime Script RT Master Mix (ESscience, Shanghai, China). qRT-PCR was then performed with SYBR Premix Ex Taq TM (Yeasen, Shanghai, China) on Light Cycler 480 (Roche, Switzerland). The expression levels of mRNA were determined by the comparative cycle threshold (CT) (2-∆∆Ct) methods (mRNA and miRNA were normalized by GAPDH and U6, respectively). All the PCR primers were listed in Table S2.

### Cell proliferation assay

CCK8 (Yeasen, Shanghai, China) assay was performed 48 h after transfection. The A549 and H1650 cells were cultivated on a 96-well plate (1000 cells per well) for 1–6 days. SpectraMax M5 Multi-Mode Microplate Reader (Molecular Devices LLC, Sunnyvale, CA, USA) was then employed after an hour of incubation with CCK8 solution at the absorbance of 450 nm.

Cells were made into single-cell suspensions and then incubated in six-well plates at a density of 1000 cells per well for cell colony formation assay. After 2 weeks, cells were fixed in methanol and stained with 0.1% crystal violet (Weijia Biology Science and Technology Co., Guangzhou, China) for 20 min. After washed by PBS, images were captured under a microscope.

EdU proliferation assay (RiboBio, China) was conducted to detect the proliferation of the transfected cell. The cells were fixed by 4% paraformaldehyde for 20 min after being treated with 50 µM EdU for 2 h. Following Apollo staining and Hoechst33342 staining, the fluorescent microscope was applied to observe the EdU positive cells.

### Wound healing assay

This essay aimed to assess the migration abilities of cells after transfection. The cells were seeded in a six-well plate. After 24 h transfection, when the cells were dense in the field of microscopic view, three standardized wound scratches per well were generated with a sterile 20 µL pipette tip, and the 10% FBS medium was replaced with serum-free RPMI 1640. A phase-contrast microscope was employed to photograph the width of the scratch at different time frames (0,12 h and 24 h).

### Transwell assay

Migration assays were executed using a Transwell chamber with 8 µm pores (Costar, Corning, New York, NY, USA). Cells (1.0 × 10^5^) in 200 µL of serum-free RPMI 1640 medium were seeded in the upper chamber placed in the 24-well culture plate, and the lower chamber contained 700 μL medium with 10% FBS as a chemoattractant. The chambers were maintained at 37 °C and 5% CO2 for 16 h. Those invaded cells were fixed with 4% paraformaldehyde for 20 min and stained with 0.1% crystal violet (Weijia Biology Science and Technology Co., Guangzhou, China) for 30 min. A phase-contrast microscope was applied to count the number of stained cells (5 different views per well).

### Flow cytometry analysis

For the cell apoptosis, the cells were suspended by trypsin without EDTA and washed by phosphate-buffered saline (PBS) thrice. 5 µl of FITC Annexin V and 5 µl of propidium iodide (biogems, CA, USA) were immediately added to the transfected cells suspending in 500 µl of binding buffer for 15 min in dark and then examined with flow cytometry. In the cell cycle analysis, the cells seeded in 6-well-plates for 60–70% confluence was synchronized at the G0 boundary by serum-free medium for 24 h before transfection. After staying in the 70% ethanol at 4℃ overnight, the cells were stained in dark for 15 min 500 µl propidium oxide staining solution (Yishan, Technology Co., Shanghai, China), and then detected by a flow cytometer (NCEA).

### Western blot

The total proteins were collected from cells using RIPA Lysis Buffer containing 1% phenylmethylsulphonyl fluoride (PMSF) (Beyotime, Shanghai, China), and the protein concentration was measured with a BCA protein assay Kit (Beyotime, Shanghai, China). Protein were denatured at 100 °C for 8 min with DualColor Protein Loading Buffer (Life, USA). The denatured proteins were separated on 10% sodium dodecyl sulfate–polyacrylamide gel electrophoresis (SDS-PAGE) and transferred onto PVDF membranes (Millipore, Billerica, MA, USA). The membranes were incubated for 20 min in quick blocking solution (Beyotime, Shanghai, China) at room temperature followed by incubation with primary monoclonal antibodies overnight at 4 °C with soft shaking. The primary antibodies against human p53, SF3B2, SF3B3, RBM17, Lamin-B1 and GAPDH were purchased from Proteintech Group (IL, USA). SCAP was purchased from Cell Signaling Technology (Danvers, MA 01,923, USA). CDK2/4/6, Cyclin D1, SF3A3, and p53 S46 were purchased from Abcam (Cambridge, MA, USA). And enhanced chemiluminescence reagent kit (Affinity Biosciences, Jiangsu, China) was utilized for exposure after the blot incubated with secondary antibody (Proteintech) for 1 h.

### Tumor xenograft experiments

Nude mice (BALB/c, SPF grade, male, 4–5 weeks old) were obtained from Beijing Vital River Laboratory Animal Center (Beijing, China), and were maintained under specific pathogen-free conditions. All experiments were performed in accordance with the guidelines of the Institutional Animal Care and Use of SYSUCC. Mice were randomly divided into different study groups, and subcutaneously implanted with 2 × 10^7^ H1650 cells or 4 × 10^7^ A549 cells respectively. Tumor sizes were measured every 3 days using calipers, and the tumor volumes (VT) were calculated according to the formula: VT = (length) × (width)^2^/2. Finally, the mice were euthanized and the subcutaneous tumors were resected for subsequent use.

### Immunohistochemistry

Formalin‐fixed and paraffin‐embedded tissue sections were incubated with Ki67 primary antibody (dilution 1:1000; Proteintech), PCNA antibody (dilution 1:300; Proteintech), and SF3A3 (dilution 1:200; Abcam) overnight at 4 °C. Then, the sections were incubated with HRP-conjugated anti-rabbit IgG secondary antibody (dilution 1:300; Yeasen) for 2 h at room temperature. The slides were evaluated by two independent observers.

### Luciferase reporter assay

The sequence of circSCAP was cloned downstream of p-GLO Dual-Luciferase vector (Jidan, Guangzhou, China). Mutations were performed in the binding sites. The cells were cultured in a 24-well plate until showing 60–70% confluence, and then the constructed report vectors containing wild-type fragment or mutant type fragment together with renilla vector and miRNA mimics or miR-NC were co-transfected into 293-T cells using LipofectamineTM 3000 reagent. The cells were collected after 48 h for luciferase detection with the dual-luciferase reporter gene assay system (Promega, Madison, WI, USA). The firefly luciferase activity was normalized based on Renilla luciferase activity.

### p53 signaling activity assays

Si SF3A3 transfection two days later, pp53-TA-GLuc-Dura, pGLuc-Dura-TA and pySEAP reporter plasmids were transfected with addition of 10 μM pifithrin-α, and luciferase was measured 24 h later.

### RNA pull-down

The biotin-coupled RNA complex was pulled down by incubating the cell lysates with streptavidin-coated magnetic beads following the manufacturer’s instructions (Axl-bio, Guangzhou, China). The bound proteins were eluted from the packed beads and analyzed by SDS-PAGE. CircSCAP probe and control probe are shown in the Table S3. The probe solution was denatured at 90 °C for 2 min and then incubated with pre-cooled RNA structure buffer to form RNA secondary structures. Afterwards, streptavidin magnetic beads were incubated with the mixture at 250℃ for 30 min. Cells were lysed at 4℃ and centrifuged. Subsequently, the supernatant fractions were collected and mixed with the probe-bead mixture. After incubation and elution at 37℃ for 2 h, the proteins in the capture complex were identified by western blotting or mass spectrometry analysis.

### RNA immunoprecipitation (RIP) assay

RIP assay was performed using RIP-Kit (BersinBio, Guangzhou, China) according to the manufacturer’s instructions. The obtained RNA was extracted by SF3A3, human anti-Ago2 antibodies (Abcam, ab32381, Shanghai, China), or negative control IgG (Beyotime, Shanghai, China), respectively.

### RNA-FISH

Biotin-labeled antisense or sense probe for circSCAP junction and U6 were synthesized (Exon Bio, Guangzhou, China) (Table S3). Cells were incubated with 40 nM FISH probe in circRNA hybridization buffer at 37 °C for 36 h. After being washed, cells were incubated with anti-digoxin HRP conjugate at 37 °C for 1 h. Then cells were incubated with TSA (Exon Bio, Guangzhou, China) in dark for 15 min, and sealed with DAPI. The images were acquired using a fluorescence microscopy (OLYMPUS FV1000 confocal microscopy, Japan).

### Nuclear and cytoplasmic extraction

Cytoplasmic and nuclear fractions were isolated according to the manufacturer’s manual, using the reagents supplied in Invent Kit (Invent Biotechnologies, Beijing, China). Briefly, the cells were lysed in Cell Fraction Buffer on ice for 10 min. After centrifugation at 14000 rpm for 5 min at 4 °C, the supernatant was collected as cytoplasmic fraction, and then the nuclei were collected by washing the pellet with Cell Fraction Buffer.

### Immunoprecipitation (IP)

Transfected cells were lysed with Pierce™ IP Lysis Buffer (Invitrogen, CA, USA). Protein concentrations were measured with a BCA protein assay kit (Beyotime, China) and the lysates were incubated with primary antibody for Ubiquitin HA-tag (Abcam) or SF3A3 (Abcam) with protein A/G magnetic beads (MedChemExpress, USA) at 4 °C overnight. The beads were washed using IP lysis buffer for 6 times before immunobloting by the indicated antibodies.

### Statistical analysis

SPSS 20.0 (IBM, Armonk, NY, USA) and Graphpad Prism 7 (GraphPad Software Inc., CA, USA) was used for data analysis. Image J software was used to analyze cell migration, and protein expression. The data are shown as the means ± SD from at least three independent experiments. Comparisons between groups for statistical significance were performed with the two-tailed Student’s *t* test or two-way ANOVA. Paired sample* t* test and Mann–Whitney *U* test were used to compare the relative expression of circSCAP in lung tumor and peritumor tissues. Chi-square test or Fisher’s exact test was applied to study the relationship between circSCAP expression level and clinicopathological variables of lung cancer patients. The survival curves were plotted using the Kaplan–Meier method. Cox regression analyses were used to study the correlations between variables and survival. *P* < 0.05 was considered statistically significant.

## Results

### CircSCAP is down-regulated and associated with poor prognosis in lung cancer

**Fig. 1:**
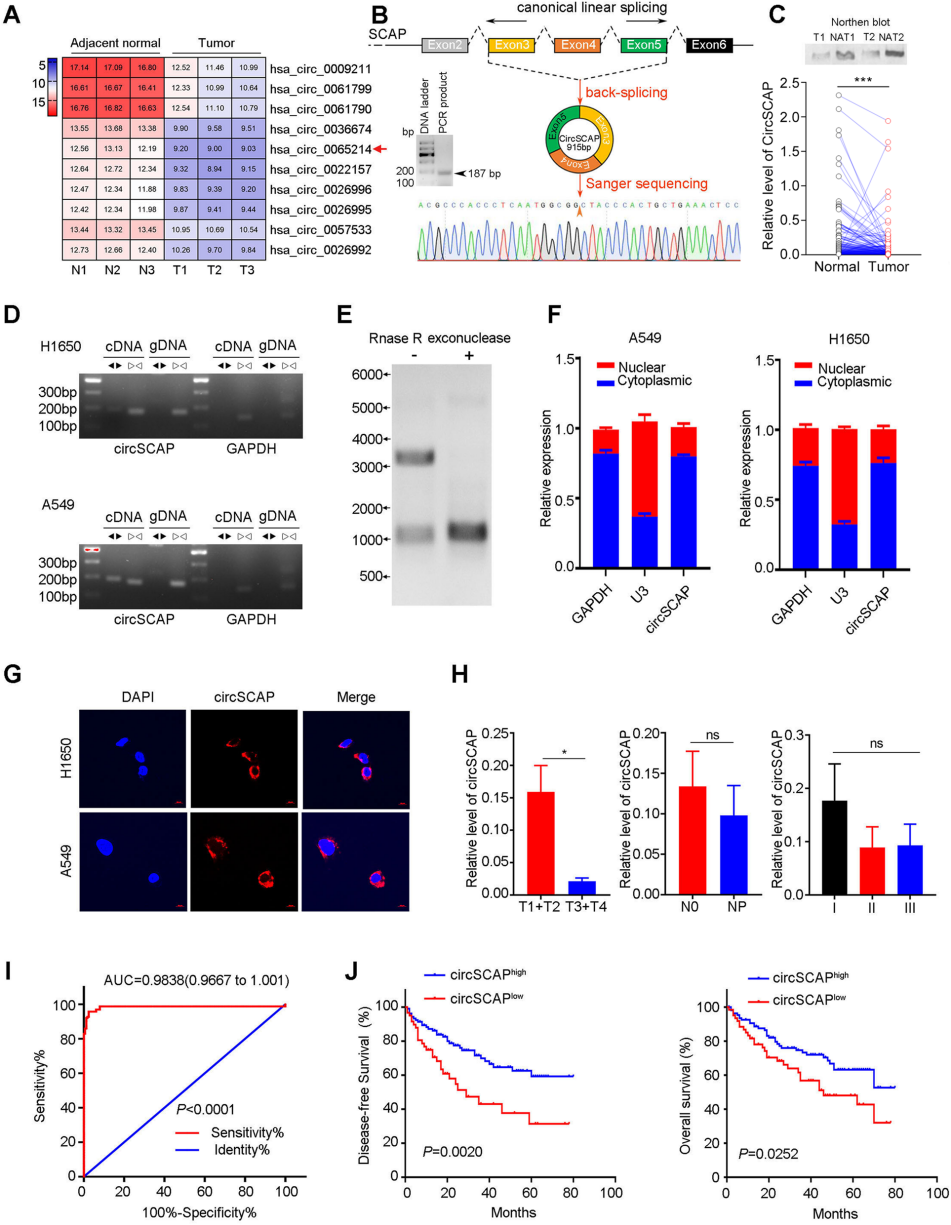
CircSCAP is down-regulated and associated with poor prognosis in lung cancer. **A** Heatmap of differentially expressed circRNAs in three pairs of human lung cancer and adjacent non-tumor tissues. The red arrow indicated the circRNA of circSCAP. **B** Schematic of circSCAP biogenesis. CircSCAP was derived from the back-splicing of the linear transcript of SCAP (the host gene). The circularization site of circSCAP was validated via PCR and sanger sequencing. **C** Northern blot analysis of circSCAP transcript level in lung cancer tissues (upper). Relative expression level of circSCAP in 161 paired normal and lung cancer tissues, as detected by RT-PCR (lower). T, tumor; NAT: normal adjacent tissue. **D** The existence of circSCAP was validated by PCR using divergent and convergent primers for cDNA and gDNA of lung cancer cell lines. **E** Northern blot analysis indicated that circSCAP was resistant to RNase R digestion. **F** CircSCAP was located in the cytoplasm of A549 (left) and H1650 (right) lung cancer cells, as shown by subcellular fractionation. **G** Representative images of circSCAP subcellular location in H1650 (upper) and A549 cell line (lower), as displayed by fluorescence in situ hybridization (FISH). **H** The expression level of circSCAP in lung cancer tissues of stage T1 + T2 was higher than that of stage T3 + T4, and the difference was statistically significant (left). No significant difference was observed between the level of circSCAP and other clinical stages (middle and right). **I** Receiver operating characteristic (ROC) curve displayed the discrimination of overall survival in lung cancer patients using circSCAP expression level. **J** Lung cancer patients with the higher level of circSCAP showed the better prognosis. Data represent the mean ± SD of triplicate experiments and were analyzed by Student t test. * means *P* < 0.05

**Fig. 2:**
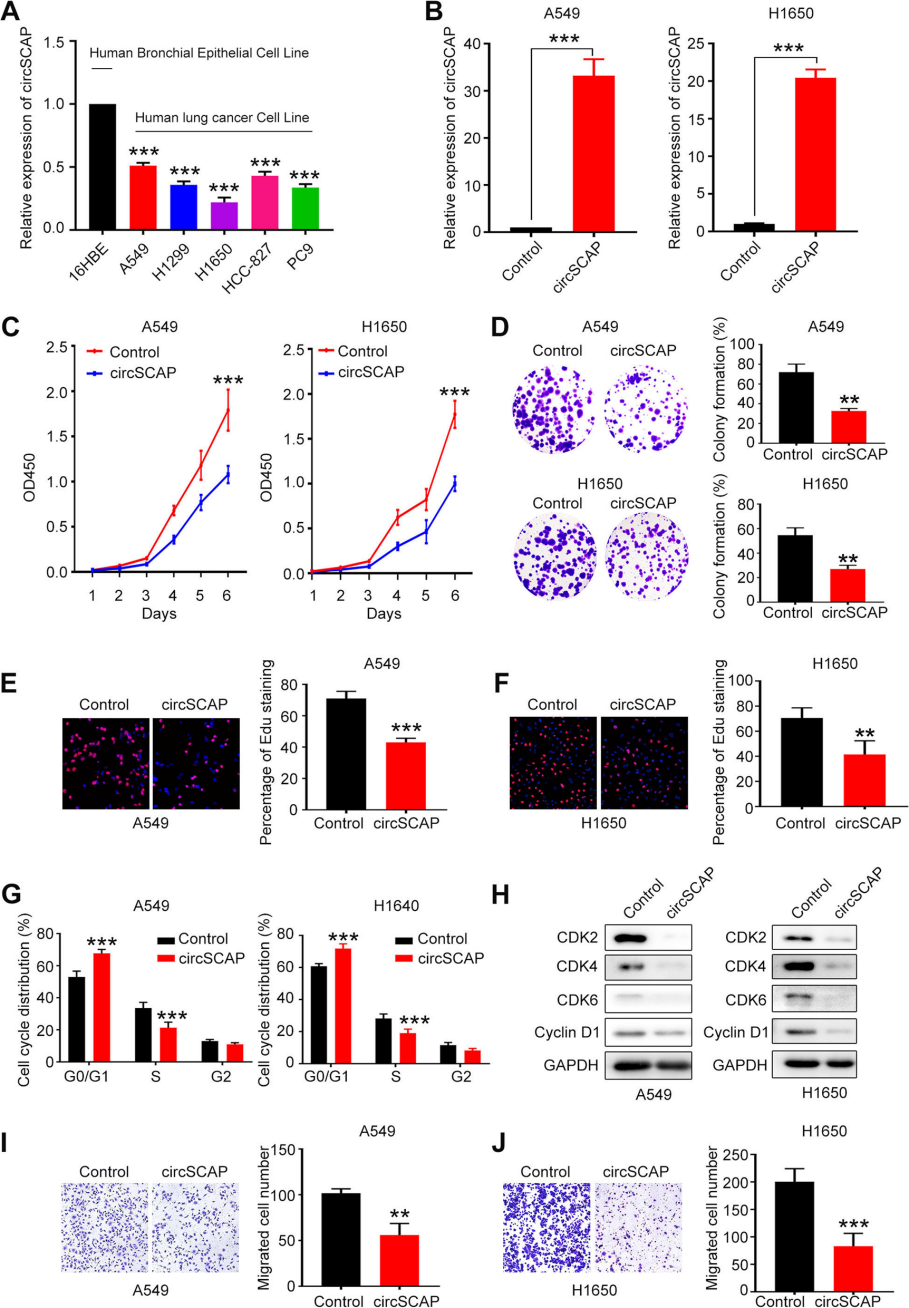
Ectopic expression of circSCAP inhibits proliferation and migration of NSCLC cells in vitro. **A** The expression level of circSCAP in five NSCLC cell lines and one normal human bronchial epithelial cell line (16HBE) was evaluated by RT-PCR. **B** CircSCAP was stably overexpressed in A549 (left) and H1650 (right) cell lines. **C-D** CCK8 and colony formation assay were performed to investigate the effect of circSCAP overexpression on cell proliferation (**C**) and colony formation ability (**D**) of NSCLC cells. **E–G** Edu staining and flow cytometry was carried out to check the cell proliferation (**E**–**F**) and cell cycle (**G**) in NSCLC cells with circSCAP overexpression compared with control. **H** The protein level of G1 checkpoints involved in cell cycle of G1 transition to G2 were tested by immunoblotting. **I-J** The migration capability of NSCLC cells stably overexpressing circSCAP were evaluated through transwell assay. Data represent the mean ± SD of triplicate experiments and were analyzed by Student t test. * means *P* < 0.05. ** means *P* < 0.01, *** means *P* < 0.001

To explore the biological function and molecular mechanisms of circRNAs in the progression of lung cancer, we first used circRNA microarray to identify differentially expressed circRNAs (DEcircRNAs) in three pairs of non-small cell lung cancer and peritumor tissues. A total of 810 DEcircRNAs were discovered (fold change > 2.0 and *P* < 0.05). Among them, 178 circRNAs were upregulated and 632 were downregulated in tumor tissues (Figure S1A). Given the expression level and statistical significance of DEcircRNAs in lung tumors, 10 candidate DEcircRNAs were identified (Fig. 1A). The expression level of top 5 candidate DEcircRNAs was next evaluated in five lung cancer cell lines, among which, hsa_circ_0065214 displayed the completely concordant alteration in microarray data and five lung cancer cell lines (Figure S1B and Fig. 2A) and thus selected for further study. The circRNA hsa_circ_0065214 derived from its host gene SCAP (collectively circSCAP, Fig. 1B) was markedly downregulated in lung cancer. Next, to investigate whether all the circRNAs originated from SCAP were downregulated in lung cancer, we searched the circBase database and found that four circRNAs derived from SCAP (hsa_circ_0065201, hsa_circ_0065214, hsa_circ_0065215, and hsa_circ_0065217) were detectable in lung cancer cell line A549. The relative expression level of the four circRNAs in lung cancer cells and lung cancer tissues suggested only hsa_circ_0065214 was specifically downregulated (Figure S1C-D). The downregulation of circSCAP was further validated in 161 paired lung tumor and peritumor tissues (Fig. 1C, *p* < 0.001) by RT-PCR and northern blot. The circularization of circSCAP was confirmed through sanger sequencing (Fig. 1B lower), PCR using divergent and convergent primers (Fig. 1D), RNase R digestion assay (Fig. 1E). Subcellular location of circSCAP was explored using RT-PCR for cytoplasmic and nuclear RNA (Fig. 1F) and RNA FISH (Fig. 1G). The results indicated circSCAP was mainly distributed in cytoplasm of lung cancer cells.

To study the relationship between circSCAP and clinical characters, we enrolled a lung cancer cohort of 173 patients. The baseline characters of the cohort were listed in Table 1. All the patients were divided into two groups according to circSCAP expression level and correlation analysis revealed that circSCAP level was negatively correlated with T stage in lung cancer (Table 2). Consistently, circSCAP level was higher in T1 + T2 than T3 + T4 stage (Fig. 1H left). The relative level of circSCAP in patients with metastasis condition and higher TNM stage was reduced but with no notable difference (Fig. 1H middle and right). Next, ROC analysis showed that circSCAP was a robust biomarker for prognosis evaluation in lung cancer (Fig. 1I). Survival analysis showed that circSCAP level was negatively associated with overall and disease-free survival (Fig. 1J). Univariate and multivariate Cox analyses indicated that circSCAP was an independent prognosis factor for overall survival of lung cancer (Table 3). Altogether, these findings suggested that circSCAP not only correlated with the progression of lung cancer but also was a promising biomarker for prognosis prediction, implying that circSCAP might play a critical role in the development and progression of lung cancer.

**Table 1:** Baseline characteristics of lung cancer patients grouped by the admission time

	**Admission time**		
**Demographics**	2013.1–2013.12	2014.1–2014.12	*P*
**Number (n)**	81	92	
**Age (y)**			0.001
≤ 60	20(24.7)	47(51.1)	
> 60	61(75.3)	45(48.9)	
**Gender**			**0.504**
Male	26(32.5)	25(27.2)	
Female	54(67.5)	67(72.8)	
**Histological Type**			0.710
SCC	40(49.4)	42(45.7)	
Adenocarcinoma	25(30.9)	27(29.3)	
Other	16(19.8)	23(25.0)	
**Expression level of circ SCAP**^**a**^			0.085
Low	35(43.2)	28(30.4)	
High	46(56.8)	64(69.6)	
**T stage**			0.184
T1 + T2	61(75.3)	60(65.2)	
T3 + T4	20(24.7)	32(34.8)	
**N stage**			0.489
N0	42(51.9)	52(56.5)	
N1	16(19.8)	12(13.0)	
N2	23(28.4)	28(30.4)	
**Grade**			0.450
G1	15(18.5)	11(12.0)	
G2	24(29.6)	27(29.3)	
G3	42(51.9)	54(58.7)	
**TNM stage**			0.199
I	28(34.6)	26(28.3)	
II	26(32.1)	23(25.0)	
III	27(33.3)	43(46.7)	
**Vessel invasion**			1.000
No	60(74.1)	69(75.0)	
Yes	21(25.9)	23(25.0)	
**Recurrence or metastasis**			0.159
No	47(58.0)	63(68.5)	
Yes	34(42.0)	29(31.5)	
**Postoperative CRT**			0.129
No	31(38.3)	46(50.0)	
Yes	50(61.7)	46(50.0)	

^a^The Optimal cutoff point of circSCAP expression was confirmed by the ROC curve

Data are mean ± SD or n (%)

*SCC* squamous cell carcinoma, *CRT* chemoradiation therapy, G1 = well differentiated, G2 = moderately differentiated, G3 = poorly differentiated

**Table 2:** The relationship between circSCAP expression and the clinicopathological parameters of lung cancer patients

	circSCAP expression^a^		
**Demographics**	Low	High	*P*
**Number (n)**	63	110	
**Age (y)**			1.000
≤ 60	24(38.1)	43(39.1)	
> 60	39(61.9)	67(60.9)	
**Gender**			**0.059**
Male	50(79.4)	72(65.5)	
Female	13(20.6)	38(34.5)	
**Histological Type**			0.545
SCC	22(34.9)	30(27.3)	
Adenocarcinoma	27(42.9)	55(50.0)	
Other	14(22.2)	25(22.7)	
**T stage**			**0.040**
T1 + T2	38(60.3)	83(75.5)	
T3 + T4	25(39.7)	27(24.5)	
**N stage**			0.477
N0	31(49.2)	63(57.3)	
N1	10(15.9)	18(16.4)	
N2	22(34.9)	29(26.4)	
**Grade**			0.603
G1	9(14.3)	17(15.5)	
G2	16(25.4)	35(31.8)	
G3	38(60.3)	58(52.7)	
**TNM stage**			0.471
I	19(30.2)	36(31.8)	
II	15(23.8)	34(30.9)	
III	29(46.0)	41(37.3)	
**Vessel invasion**			0.329
No	37(58.7)	73(66.4)	
Yes	26(41.3)	37(33.6)	
**Recurrence or metastasis**			0.166
No	25(56.8)	44(63.8)	
Yes	19(43.2)	25(36.2)	
**Postoperative CRT**			0.753
No	27(42.9)	50(45.5)	
Yes	36(57.1)	60(54.5)	

^a^The optimal cutoff point of circSCAP expression was confirmed by the ROC curve

Data are mean ± SD or n (%)

*SCC* squamous cell carcinoma, *CRT* chemoradiation therapy, G1 = well differentiated, G2 = moderately differentiated, G3 = poorly differentiated

**Table 3:** Cox regression analysis of prognostic factors influencing overall survival

**Variables**	**Univariate analysis**			**Multivariate analysis**		
	HR	95% CI	*P*	HR	95% CI	*P*
**Age (y)**						
≤ 60	1			-		
> 60	1.114	0.663–1.870	0.684			
**Gender**						
Female	1			-		
Male	1.640	0.888–3.029	0.114			
**T stage**						
T1	1			1		
T2	1.731	0.716–4.186	0.223	1.703	0.703–4.128	0.239
T3	3.571	1.416–9.009	**0.007**	3.609	1.410–9.235	**0.007**
T4	2.616	0.925–7.398	0.070	2.937	1.030–8.373	**0.044**
**N stage**						
N0	1			1		
N1	2.103	1.049–4.218	**0.036**	2.371	1.172–4.798	**0.016**
N3	2.060	1.173–3.616	**0.012**	1.831	1.020–3.287	**0.043**
**Grade**						
G1	1			-		
G2	0.903	0.374–2.179	0.820			
G3	1.471	0.689–3.144	0.319			
**TNM stage**						
I	1			-		
II	1.212	0.567–2.590	0.620			
III	2.235	1.156–4.320	**0.017**			
**Vessel invasion**						
No	1			-		
Yes	1.306	0.753–2.266	0.343			
**Adjuvant therapy**						
No	1			-		
Yes	0.836	0.499–1.402	0.498			
**Expression level of circSCAP**						
Low	1			1		
High	0.585	0.353–0.970	**0.038**	0.636	0.380–0.984	**0.048**

*HR* hazard ratio, *CI* confidence interval

### CircSCAP inhibits proliferation and migration ability but promotes apoptosis of non-small cell lung cancer cells in vitro

**Fig. 3:**
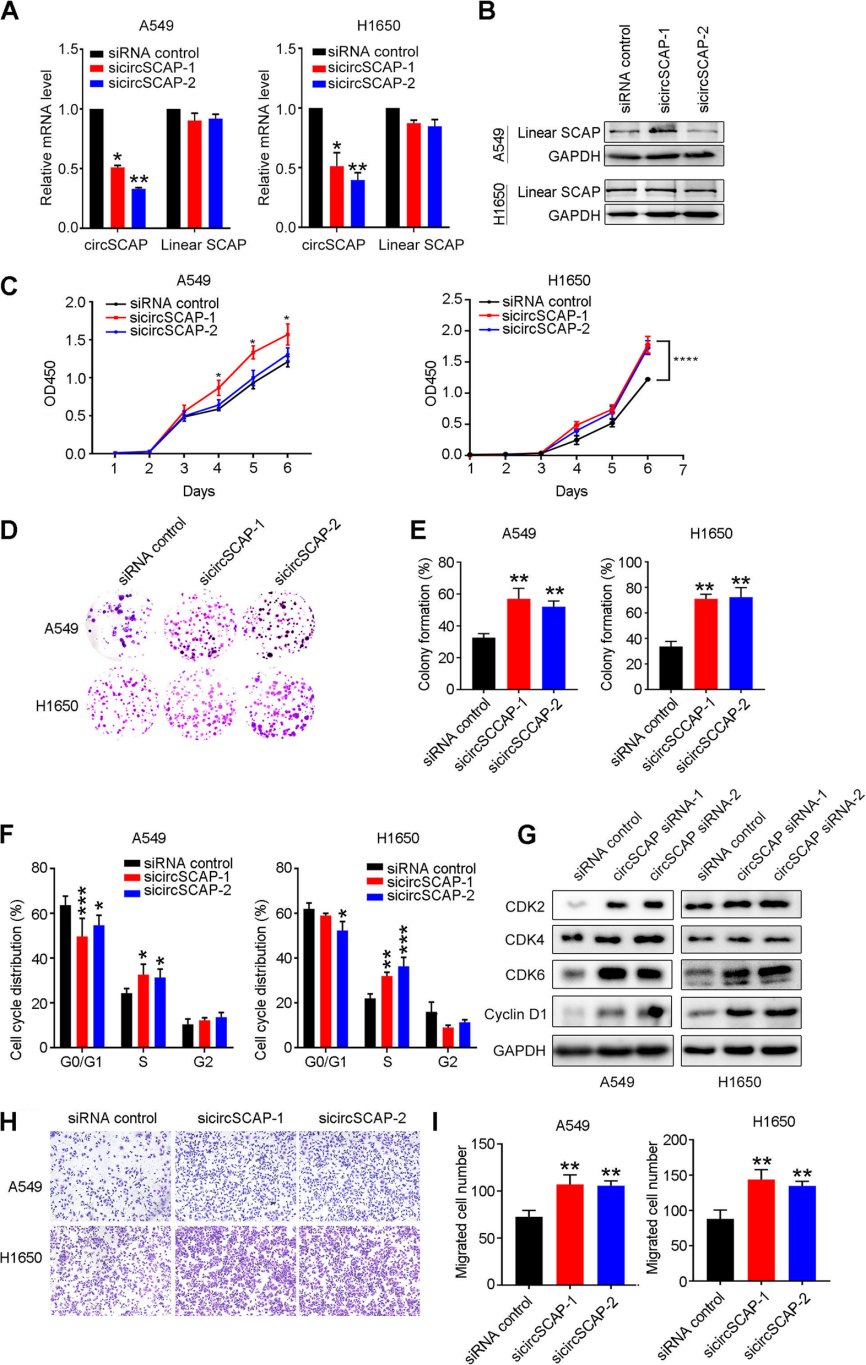
Knock-down of circSCAP promotes proliferation and migration of NSCLC cells in vitro. **A** RT-PCR was performed to verify the knock-down efficiency of two circSCAP siRNAs. **B** Western blot indicated that circSCAP decrease exerted no effect on the level of linear SCAP (host gene of circSCAP). **C**-**E** CCK-8 and colony formation assays were performed to determine the proliferation (**C**) and colony formation ability (**D**-**E**) of NSCLC cells with circSCAP knock-down. **F** Flow cytometry was performed to check the effect of circSCAP decrease on cell cycle of NSCLC cells. **G** The protein level of G1 checkpoints involved in cell cycle of G1 transition to G2 were tested by immunoblotting. **H**-**I** Transwell assay was performed to determine the migration ability of NSCLC cells with circSCAP knock-down when compared with control. Data represent the mean ± SD of triplicate experiments and was analyzed by Student t test. * means *P* < 0.05. ** means *P* < 0.01, *** means *P* < 0.001

Non-small cell lung cancer (NSCLC) accounts for the majority of lung cancer diagnosis (about 80% in the cohort used in this study). Therefore, we focused the role of circSCAP in NSCLC in the present study. Next, to investigate the biological function of circSCAP in NSCLC, we first determined the relative level of circSCAP in NSCLC cell lines and found that circSCAP was much lower in NSCLC cells when compared to normal bronchial epithelial cell line (Fig. 2A). Among five NSCLC cell lines, A549 exhibited the highest and H1650 lowest circSCAP background level. Furthermore, A549 and H1650 were among the most commonly used cell lines for NSCLC investigation. Therefore, we chose A549 and H1650 cell lines for further investigation (Fig. 2A). Then we established NSCLC cells with circSCAP overexpression (Fig. 2B) or knock-down (Fig. 3A), as validated by the RT-PCR. We also confirmed that the overexpression (Figure S2A) or knock-down (Fig. 3B) of circSCAP showed no significant effect on the expression level of linear SCAP. After that, the proliferation and colony formation capacity of NSCLC cells with circSCAP overexpression or knock-down was explored by CCK8 (Figs. 2C and 3C), colony formation assay (Figs. 2D and 3D-E) and Edu staining (Fig. 2E and F). The results showed that high level of circSCAP significantly inhibited while low level promoted the proliferation, colony formation ability of NSCLC cells. The proliferation ability of cancer cells was closely related to cell cycle process. Hence, we further determined the effect of circSCAP on cell cycle of NSCLC cells and found that circSCAP markedly block the process of G1 transition to S phase of cell cycle (Figs. 2G and 3F). We next checked the key G1 phase cell cycle checkpoints (CDK2, CDK4, CDK6 and Cyclin D1) by immunoblotting and found that these checkpoints were expectedly downregulated by circSCAP (Figs. 2H and 3G).

Furthermore, we tested the role of circSCAP in the migration of NSCLC cells and found that circSCAP overexpression notably inhibited (Fig. 2I-J and Figure S2B-C) while knock-down promoted (Fig. 3H-I and Figure S2D-E) the migration ability of NSCLC cells, as displayed by transwell and wound healing assay. Besides, we also detected whether circSCAP affected apoptosis of NSCLC cells. As expected, the NSCLC cells overexpressing circSCAP (Figure S2F and G) suffered boosted apoptosis while cells with circSCAP knock-down (Figure S2H and I) exhibited lowered apoptosis. Taken together, these results above demonstrated that circSCAP exerted tumor-suppressing functions in vitro.

### Ectopic expression of circSCAP inhibits NSCLC malignance in vivo

**Fig. 4:**
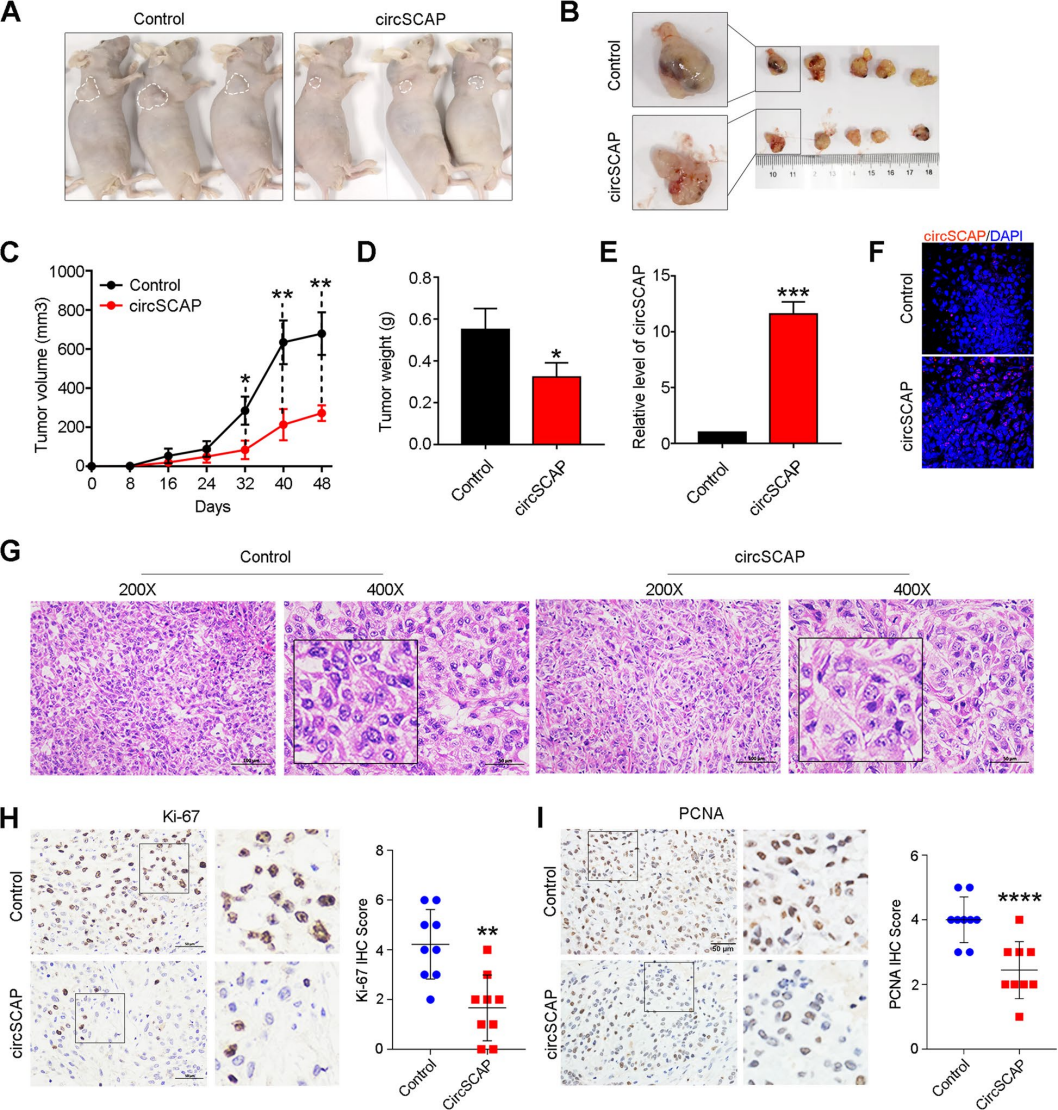
Ectopic expression of circSCAP inhibits the proliferation of NSCLC cells in vivo. **A** Representative images of subcutaneous xenograft tumors of H1650 cells with circSCAP overexpressing or empty vector control (*n* = 5 for each group). **B** Representative images showed tumor morphology of xenograft in nude mice. **C**-**D** Ectopic expression of circSCAP suppressed the tumor growth (**C**) and decreased tumor weight (**D**) of xenograft tumors. **E–F** RT-PCR (**E**) and FISH (**F**) assay of the tumor tissues captured at the end point indicated the circSCAP was overexpressed in xenograft tumor. **G** Representative images of HE staining showed the pathological configuration of xenograft tumors (left: magnification, 200 × ; right: magnification, 400 ×). **H**-**I** Ki-67 (**H**) and PCNA (**I**) IHC staining showed the proliferation capacity of H1650 cells stably overexpressing circSCAP when compared with control cells in xenografts. * means *P* < 0.05. ** means *P* < 0.01, *** means *P* < 0.001

To further investigate the tumor-suppressing function of circSCAP in vivo, stable H1650 cell line was built by infection with lentivirus carrying circSCAP overexpression or control vector. These cells were injected subcutaneously into flank of nude mice and tumor growth curves were recorded. Expectedly, we found circSCAP overexpression significantly suppressed the growth of H1650 cells (Fig. 4A-C) and mice bearing circSCAP overexpressing cells showed lighter tumor weight compared to that of control mice (Fig. 4D). The circSCAP was ascertained to highly expressed in xenografts by RT-PCR (Fig. 4E) and RNA FISH (Fig. 4F). The xenografts were confirmed to be NSCLC tumors in hematoxylin and eosin (H&E) staining slides under a microscope by an experienced pathologist (Fig. 4G). Additionally, immunohistochemical (IHC) staining of Ki-67 and PCNA in xenografts showed a significant decrease of the positive intensity (IHC score) in the circSCAP overexpression group (Fig. 4H-I). Collectively, we confirm that ectopic expression of circSCAP inhibits NSCLC progression in vivo.

### CircSCAP directly sponges miR-365b-3p and binds to SF3A3 protein

The above results indicate that circSCAP inhibits the malignance of NSCLC cells in vitro and in vivo, while the underlying molecular mechanism remains to be elucidated. Generally, the subcellular distribution of circRNAs affects its function. Previous studies reported that circRNAs located in the cytoplasm mainly serve as competing endogenous RNAs (ceRNAs) and that in nucleus mainly function through binding proteins [19–22]. Here, circSCAP was mainly located in the cytoplasm (Fig. 1E and F). Therefore, we first investigated whether circSCAP served as the microRNAs sponge to function in NSCLC cells. Four candidate microRNAs (miR-365b-3p, miR-15a-5p, miR-15b-5p and miR-16-5p) most likely sponged by circSCAP were obtained by intersection of microRNA sequencing analysis and bioinformatics prediction (Figure S3A). Luciferase reporter assay indicated that circSCAP owned binding capacity for all the four microRNAs (Figure S3B), but RIP-PCR assay suggested only miR-365b-3p could be enriched by circSCAP on AGO2 protein (Figure S3C) implying circSCAP likely function through sponging miR-365b-3p. Not to be expected, the level of miR-365b-3p in lung cancer showed no significant difference when compared to that of peritumor tissues (Figure S3D) and miR-365b-3p inhibited the proliferation of NSCLC cells (Figure S3E and F), which was opposite to the expected results. Moreover, miR-365b-3p exerted no effect on the colony formation ability of NSCLC cells (Figure S3G and H). Taken together, circSCAP could directly sponge miR-365b-3p but not function through it, indicating circSCAP likely functions via binding proteins.

**Fig. 5:**
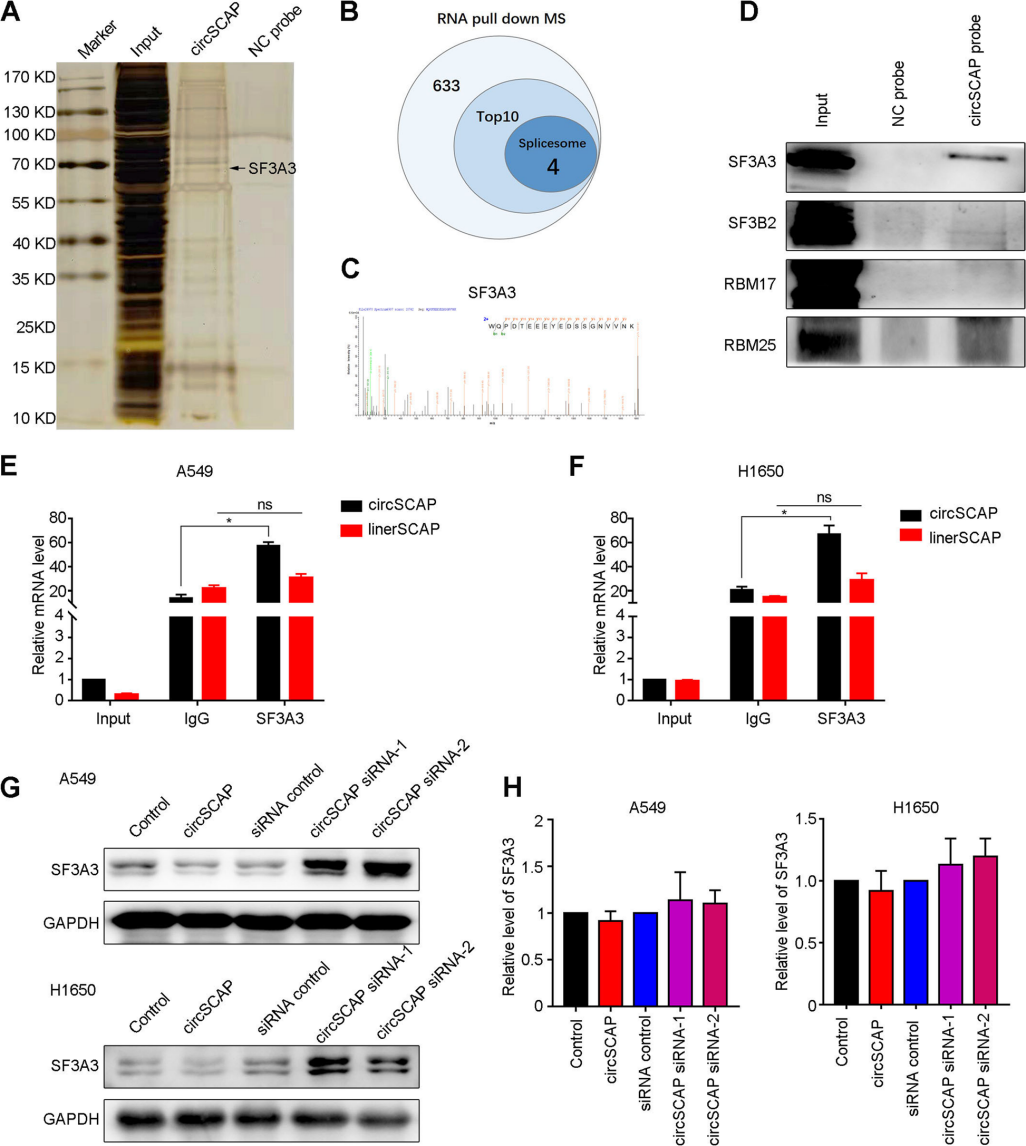
CircSCAP directly interacts with SF3A3 protein. **A** CircSCAP binding proteins were pulled down via circSCAP probe in A549 cells and displayed by silver staining. **B** All precipitated complex pulled down by circSCAP and negative control probe were subjected to mass spectrum analysis. A total of 633 proteins were enriched for circSCAP probe when compared with negative control, among which 4 of top 10 proteins were involved in splicesome. **C**-**D** Top 4 candidate proteins of splicesome pulled down by circSCAP probe were validated via immunoblotting (**D**). CircSCAP directly interacted with SF3A3 protein, as shown by mass spectrum (**C**) and immunoblotting (**D**) in A549 cells. **E**–**F** RNA Immunoprecipitation (RIP) assay was performed to confirm the interaction of circSCAP and SF3A3 protein in A549 (**E**) and H1650 cells (**F**). **G** CircSCAP overexpression significantly decreased while knock-down enhanced SF3A3 protein level in NSCLC cell lines. **H** CircSCAP level showed no effects on the mRNA level of SF3A3. Data represent the mean ± SD of triplicate experiments and were analyzed by Student t test. * means *P* < 0.05

To identify the candidate proteins directly interacting with circSCAP, we carried out mass spectrum analysis for the pulled down complexes by circSCAP and control probes. Silver staining showed the specific proteins pulled down by circSCAP probe (Fig. 5A). A total of 633 proteins were enriched for circSCAP probe when compared with negative control (Additional file 15). Meanwhile, gene sets enrichment analysis (GSEA) for the differentially expressed genes in circSCAP overexpression NSCLC cells versus control cells revealed that circSCAP inhibited the spliceosome pathway (Figure S4A), suggesting that circSCAP might bind proteins related to spliceosome. As expected, we found four splicing factors (SF3A3, SF3B2, RBM17, RBM25) involved in spliceosome pathway in the top 10 proteins specifically pulled down by circSCAP probe (Fig. 5B and C). Further immunoblotting validation (Fig. 5D) for the pull-down complexes and RIP-PCR validation (Fig. 5E and F) for the immunoprecipitated RNA showed that only SF3A3 could be directly bound by circSCAP. Next, we wanted to know whether circSCAP affected the expression level of SF3A3. To this purpose, we performed immunoblotting and RT-PCR analysis for the NSCLC cells with circSCAP overexpression or knock-down and found that circSCAP could reduce SF3A3 protein level (Fig. 5G) but not mRNA level (Fig. 5H), indicating circSCAP might regulate SF3A3 expression in a posttranscriptional manner.

### CircSCAP promotes SF3A3 degradation via ubiquitin-mediated proteasomal pathway

**Fig. 6:**
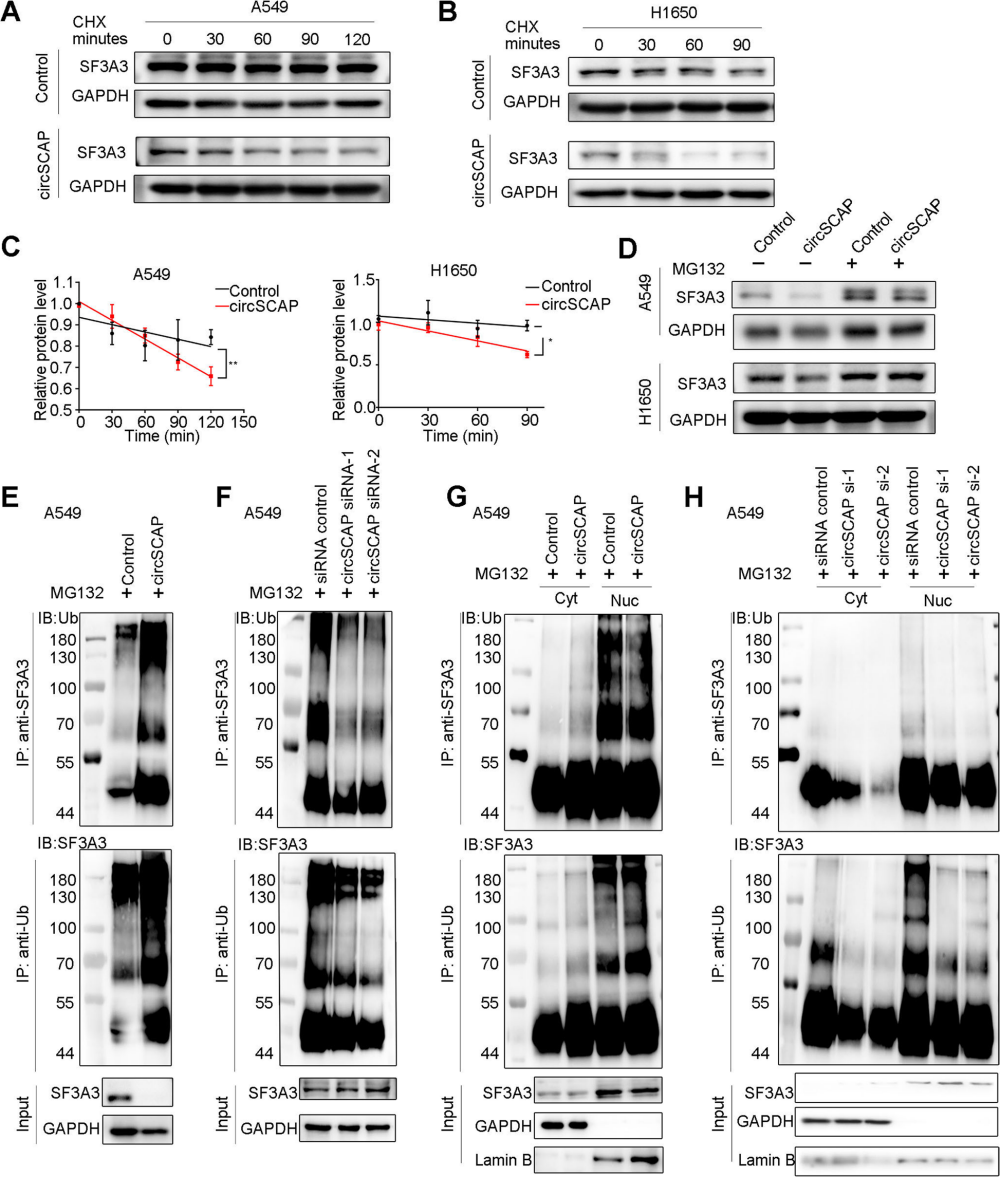
CircSCAP downregulates SF3A3 protein level by promoting the ubiquitination-proteasome pathway. **A**-**B** NSCLC cells (A549 and H1650) with circSCAP overexpression and control vector were treated with cycloheximide (CHX; 20 μg/ml) for the indicated times followed by immunoblotting to determine the protein stability of SF3A3. **C** CircSCAP overexpression notably prolonged the half-life of SF3A3 in A549 and H1650 cells. **D** Higher level of circSCAP in A549 and H1650 led to decrease of SF3A3 protein level which was restored by MG132 treatment (10 μM) for 24 h, as shown by immunoblotting. **E**–**F** A549 cells with circSCAP overexpression (**E**) or knock-down (**F**) were treated with MG132 (10 μM) for 24 h, and then cell lysates were immunoprecipitated with SF3A3 antibody and the precipitated complexes were subjected to western blot with ubiquitin-HA antibody (upper); or the cell lysates were immunoprecipitated with ubiquitin-HA antibody and the precipitated complexes were subjected to western blot with SF3A3 antibody (lower). The experiments were aimed to detect the ubiquitination status of SF3A3 protein after overexpression or knock-down of circSCAP. **G**-**H** A549 cells with circSCAP overexpression (**G**) or knock-down (**H**) were treated with MG132 (10 μM) for 24 h, and then cytosolic and nuclear components of cell lysates were separated and subjected to the same immunoprecipitation and immunoblotting as in E–F. * means *P* < 0.05. ** means *P* < 0.01

Given that circSCAP only affected SF3A3 protein level rather than mRNA level, we speculated that circSCAP likely influenced the half-life of SF3A3 protein. To validate our surmise, we treated NSCLC cells stably overexpressing circSCAP or control vector with cycloheximide (CHX) for the indicated times followed by immunoblotting. We found that the half-life of SF3A3 protein in NSCLC cells with circSCAP overexpression was much shorter than that of control cells (Fig. 6A-C), implying that circSCAP could weaken the stability of SF3A3 protein. In general, protein stability was closely related to proteasome. Therefore, we further explored whether proteasome mediated circSCAP-driven instability of SF3A3. As expected, the decrease of SF3A3 protein level upon circSCAP overexpression was rescued by MG132 treatment, as exhibited by immunoblotting (Fig. 6D), suggesting that circSCAP downregulated SF3A3 protein level through proteasome-mediated degradation.

As we have well known, proteasome is an organelle mainly degrading the ubiquitin-labelled proteins [23]. Hence, we continued to ask whether circSCAP affected the ubiquitination of SF3A3 protein and then promoted its proteasome-mediated degradation. To this end, we tested the level of ubiquitin-labeled SF3A3 by immunoprecipitation using SF3A3 or ubiquitin antibody in NSCLC cells. The results showed that the ubiquitin level of SF3A3 was significantly increased upon circSCAP overexpression (Fig. 6E and Figure S5A) while decreased upon circSCAP knock-down (Fig. 6F and Figure S5B). Consistently, GSEA result also showed that the ubiquitin protein specific protease activity pathway was significantly enriched in NSCLC cells overexpressing circSCAP (Figure S4B). Since circSCAP was mainly located in cytoplasm (Fig. 1F and G) but SF3A3 in nuclear of NSCLC cells (Figure S6B), we want to know how circSCAP affected the ubiquitination of SF3A3. Therefore, we further detected the level of ubiquitin-labeled SF3A3 in cytoplasmic and nuclear components separately and found that circSCAP overexpression obviously enhanced the ubiquitination of SF3A3 in cytoplasm but not nucleus (Fig. 6G and Figure S5C). Correspondingly, circSCAP knock-down notably reduced the ubiquitination of SF3A3 in cytoplasm of two NSCLC cell lines but no consistent results in nucleus of the two cell lines (Fig. 6H and Figure S5D). Collectively, the results above implied circSCAP mainly promoted the ubiquitin-mediated proteasomal degradation of SF3A3 in cytoplasm by directly binding to SF3A3 before its translocation from cytoplasm to nuclei, which was consistent with the subcellular location of circSCAP.

### CircSCAP inhibits the malignance of NSCLC by downregulating SF3A3

Previous studies reported that SF3A3 was upregulated and acted as an oncogenic protein in liver cancer [24]. However, the biological function of SF3A3 in NSCLC remains elusive. In this study, we found circSCAP could promote the degradation of SF3A3 in NSCLC cells and whether circSCAP exerted tumor-suppressing function through downregulating SF3A3 needed to be investigated. We first determined the mRNA level of SF3A3 in lung cancer and found that SF3A3 was significantly upregulated in lung cancer compared to peritumor tissues (Figure S6A). Consistently, the protein level of SF3A3 in lung cancer was also obviously elevated in lung cancer, as shown by IHC staining (Figure S6B). Survival analysis revealed that high SF3A3 mRNA level predicted bad prognosis of lung cancer (Figure S6C, log rank = 6.402, *p* = 0.0093). These results implied that SF3A3 likely served as an oncogenic protein in NSCLC. To confirm the tumor-promoting function of SF3A3 in NSCLC, we performed gain and loss of SF3A3 function in vitro. The SF3A3 level was significantly overexpressed and knocked down in NSCLC cells (Figure S6D-E and H-I). In vitro assay suggested, SF3A3 overexpression could notably promote but knock-down suppress the malignance including proliferation (Figure S6F and J) and colony formation ability (Figure S6G and K-L) of NSCLC cells. Taken together, these results proved that SF3A3 functioned as an oncogenic protein in NSCLC.

**Fig. 7:**
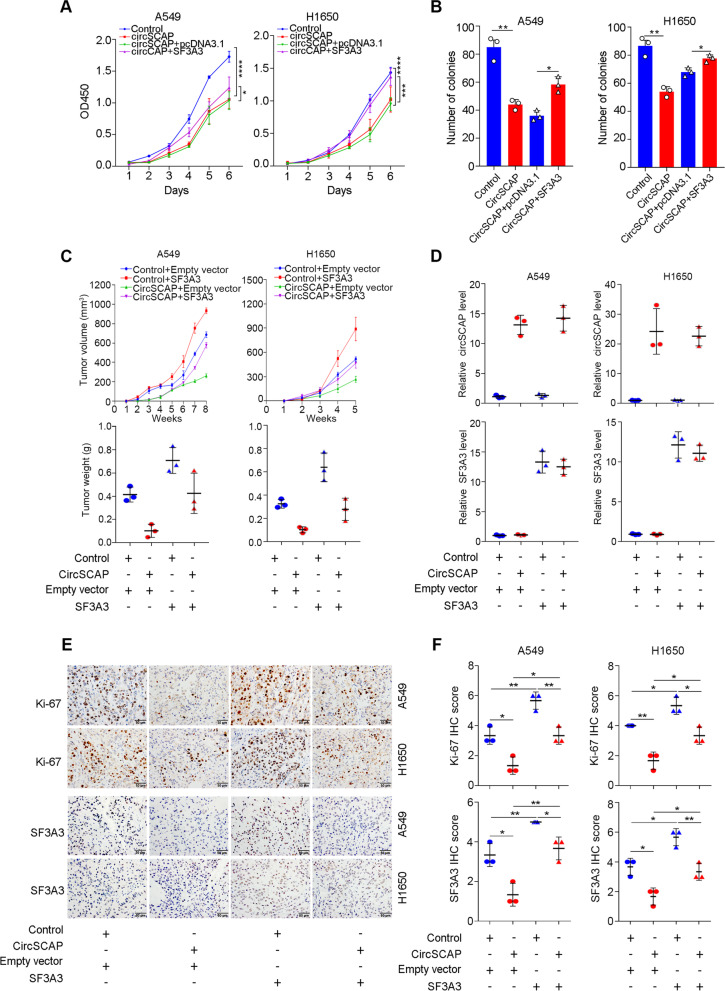
CircSCAP inhibits the malignance of NSCLC by downregulating SF3A3 in vitro and in vivo. **A**-**B** NSCLC cells with circSCAP stable overexpression exhibited weakened proliferation (**A**) and colony formation ability (**B**) when compared with control, while SF3A3 could rescue the phenotype, as shown by CCK8 and colony formation assay. **C** In vivo growth curve (upper panel), and weight at the end points (lower panel) of xenograft tumors formed by subcutaneous injection of NSCLC cells co-transfected with circSCAP and/or SF3A3 into the dorsal flanks of nude mice (*n* = 3 for each group). **D** Relative expression level of circSCAP and SF3A3 in subcutaneous xenografts of nude mice with the indicated treatment were determined via RT-PCR. **E**–**F** Representative image (**E**) and quantification (**F**) of immunohistochemical staining showing the expression of Ki-67 and SF3A3 of xenografts (*n* = 3 for each group). Scale bars: 50 μm. * means *P* < 0.05. ** means *P* < 0.01, *** means *P* < 0.001

To further investigate whether circSCAP exhibited tumor-suppressing function by downregulating SF3A3 in NSCLC, we carried out rescue assay in vitro and in vivo. As expected, circSCAP overexpression inhibited the malignance of NSCLC cells including the proliferation (Fig. 7A) and colony formation ability (Fig. 7B and Figure S7), but these phenotypes could be rescued by subsequent SF3A3 supplementation in vitro (Fig. 7A-B and Figure S7). Furthermore, NSCLC cells with circSCAP overexpression followed by SF3A3 supplementation were subcutaneously injected into flank of nude mice for in vivo assay. Consistent with the in vitro results, we found that SF3A3 supplementation successfully reversed the tumor-suppressing function of circSCAP overexpression, as displayed by the tumor volume (Fig. 7C upper and Figure S8A) and tumor weight (Fig. 7C lower) alteration. The xenograft tumors were confirmed to be NSCLC cells in H&E staining slices by an experienced pathologist (Figure S8B) and the expected circSCAP and SF3A3 level of xenografts was also confirmed (Fig. 7D and Figure S8C). In addition, we performed IHC staining for Ki-67 and SF3A3 proteins in xenograft tumor tissue sections and found that the level of circSCAP was significantly inversely correlated to Ki-67 and SF3A3 (Fig. 7E and F), but the level of SF3A3 was positively associated with Ki-67 (Fig. 7E and F). Altogether, these data supported that circSCAP inhibited the malignance of NSCLC by downregulating SF3A3.

### SF3A3 decrease suppresses NSCLC malignance by impairing SF3A3/PRMT5 complex-mediated p53 signaling activation

**Fig. 8:**
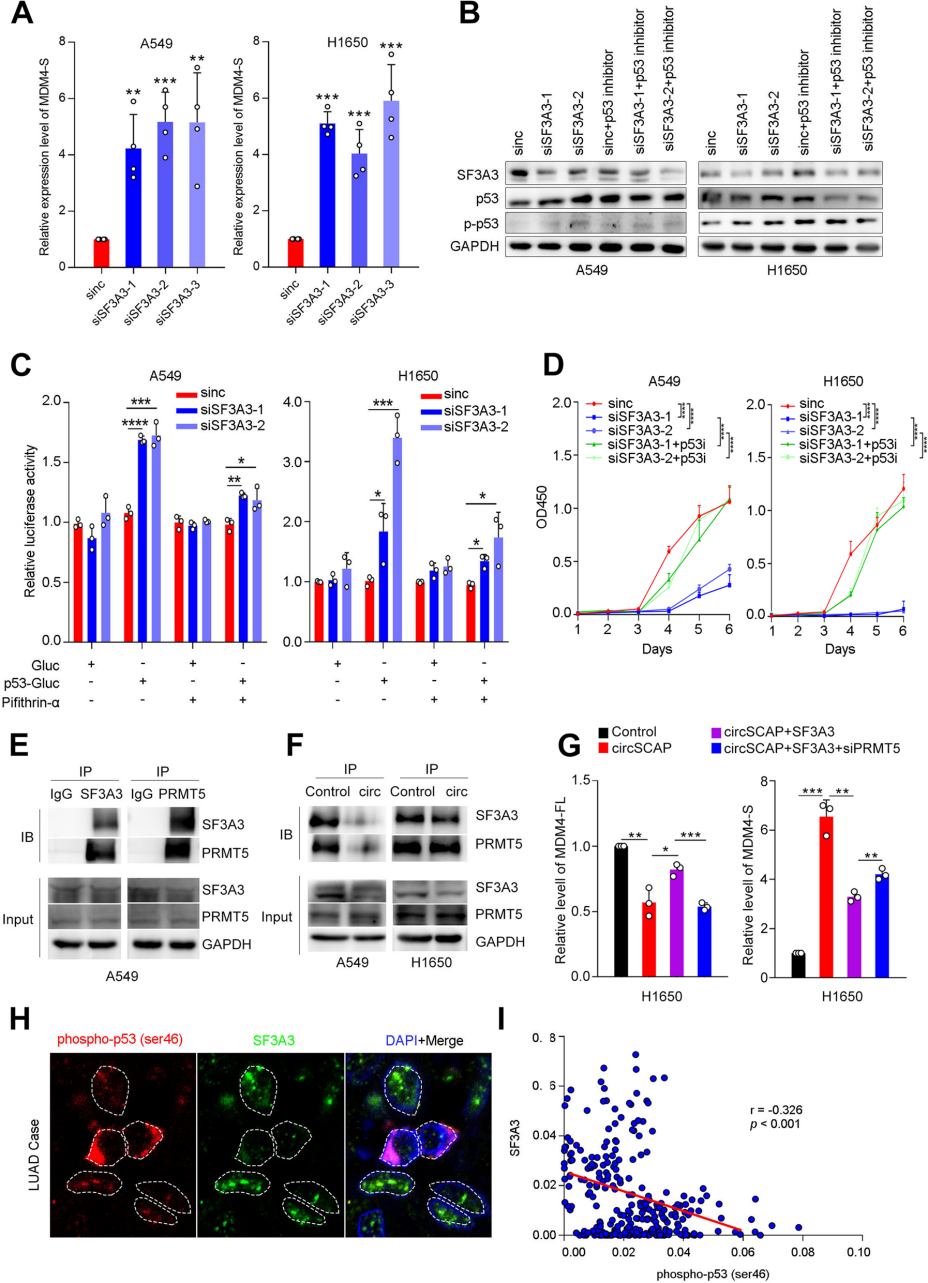
SF3A3 decrease suppressed NSCLC malignance by impairing SF3A3/PRMT5 complex-mediated p53 signaling activation. **A** The decrease of SF3A3 elevated the level of MDM4-S in A549 and H1650 as detected by RT-PCR. **B** The protein levels of SF3A3, total p53 and phospho-p53 (Ser46) in A549 and H1650 cells with the indicated treatment were assessed by immunoblotting. **C** Luciferase reporter assay indicated SF3A3 siRNAs markedly activating p53 signaling and p53 inhibitor (pifithrin-α) blunted the phenotype. **D** The proliferation of NSCLC cells with SF3A3 siRNAs transfection and/or additional treatment with 10 μM pifithrin-α was measured by CCK8 assay. **E** The direct interaction between SF3A3 and PRMT5 was confirmed in A549 cells by CO-IP assay. **F** The A549 and H1650 cells with circSCAP overexpression displayed decreased interaction between SF3A3 and PRMT5 as detected by CO-IP assay. **G** The expression level of MDM4-FL and MDM4-S of the indicated groups were determined by RT-PCR. **H**-**I** Representative confocal images of NSCLC patient tissues showed the expression of SF3A3 and phospho-p53 (Ser46) (**H**). The fluorescence intensity of SF3A3 and phospho-p53 (Ser46) was captured using image J software and the pearson correlation between them were calculated (**I**). Each dot represents the data of a single cell. * means *P* < 0.05. ** means *P* < 0.01, *** means *P* < 0.001

Next, we continued to study how SF3A3 decrease affected NSCLC cell malignance. SF3A3, as a splicing factor, has been reported to regulate the alternative splicing (AS) of MDM4 transcript [25]. As previously reported, knock-down of SF3A3 could obviously facilitate the MDM4-S (MDM4 exon 6 skipping transcript) generation [25], which was also observed in our RNA sequencing data of H1650 with SF3A3 siRNA treatment (Figure S9A). To further confirm this finding, we performed RT-PCR and found that SF3A3 siRNAs treatment markedly increased MDM4-S level (Fig. 8A). MDM4-S is a variant derived from MDM4 splicing and encodes a truncated protein that contains the p53-binding domain [26, 27]. Moreover, MDM4 was reported as an important p53 regulator [27]. Based on these findings, we hypothesized that SF3A3 decrease likely exhibited tumor-suppressing function by regulating p53 signaling.

We hence further determined the effect of SF3A3 knock-down on p53 signaling. As expected, decrease of SF3A3 notably enhanced the level of total p53 and phosph-p53 (ser 46) while pifithrin-α (a p53 signaling inhibitor) reversed the phenotype (Fig. 8B). Luciferase reporter assay (p53 transcriptional activity reporter vector) showed that decrease of SF3A3 could significantly activating p53 signaling while pifithrin-α reversed it (Fig. 8C and Figure S9B). Furthermore, we carried out rescue assay in vitro including CCK8 and cell cycle test. The results indicated that p53 signaling inhibitor could obviously rescued the proliferation repression and G1 phase blockade resulted from SF3A3 downregulation (Fig. 8D and Figure S9C). Next, we aimed to reveal how SF3A3 affected the level of MDM4-S. As reported, the precursor RNA of MDM4 could be spliced to MDM4-FL and MDM4-S, and the process was regulated by protein arginine methyltransferase 5 (PRMT5). As is well documented, the loss of PRMT5 will weaken the splicing from MDM4 precursor to MDM4-FL but enhance splicing to MDM4-S. And in the present study, we found that the decrease of SF3A3 also increased the generation of MDM4-S. In addition, the protein database (String) revealed that the protein SF3A3 could directly bind to PRMT5. Thus, we speculated that SF3A3 affected the level of MDM4-S by forming a complex with PRMT5, which regulated the splicing of MDM4 precursor RNA to MDM4-FL or MDM4-S. Expectedly, the decrease of SF3A3 could reduce the MDM4 precursor RNA splicing to MDM4-FL but enhance splicing to MDM4-S (Figure S9D and Fig. 8A). In addition, in NSCLC cells we confirmed that SF3A3 could directly bind to PRMT5 (Fig. 8E and Figure S9E) and circSCAP weakened the SF3A3/PRMT5 complex formation by decreasing the protein level of SF3A3 but not PRMT5 (Fig. 8F), which was further validated when SF3A3 was downregulated by circSCAP overexpression and siRNAs treatment (Figure S9F and G). To ascertain SF3A3 regulate the level of MDM4-S through PRMT5, we performed the rescue assay and the results indicated that SF3A3 inhibited the MDM4 precursor RNA splicing to MDM4-S through PRMT5 (Fig. 8G and Figure S9H). Together, we concluded that SF3A3 affected the level of MDM4-S by forming a complex with PRMT5. To further confirm our finding that circSCAP exerted tumor-suppressing function via SF3A3/p53 signaling, we performed immunofluorescence in NSCLC patient tumors (Fig. 8H). Correlation analysis revealed that the level of SF3A3 and phosphor-p53 (ser 46) was significantly inversely correlated (Fig. 8I, r = 0.326, *p* < 0.001). Altogether, we drew the conclusion that SF3A3 decrease suppressed NSCLC malignance by activating p53 signaling.

## Discussion

CircRNAs are expressed abundantly and play an essential role in many biological processes [28]. Moreover, aberrant expression of circRNA was demonstrated to be associated with various diseases, especially cancer [29]. Emerging studies have identified circRNAs as vital regulators in many malignancies including lung cancer [8, 28–30]. For example, circRNA 100146 affects SF3B3 expression by sponging both miR-361-3p and miR-615-5p to promote tumorigenesis of NSCLC [30]. CircFGFR1 promotes NSCLC progression and anti-PD-1 resistance by sponging miR-381-3p and upregulating CXCR4 [31]. CircTP63 increases the proliferation of lung cancer cells by sponging miR-873-3p and upregulating FOXM1-related pathway [32]. However, the function and molecular mechanism of the majority of circRNAs in lung cancer remain to be further investigated. In the current study, we first identified that circSCAP was frequently downregulated in lung cancer and circSCAP level was an independent prognosis factor for overall survival. In vitro and in vivo studies suggested that circSCAP significantly inhibited malignance of NSCLC cells. Mechanistically, circSCAP facilitated the degradation of SF3A3 leading to MDM4-S elevation resulting in p53 signaling activation.

CircRNAs have been reported to regulate transcription of their host genes [9]. In our study, we found that circSCAP did not affect the transcription of its host gene SCAP (Figure S2A and Fig. 3B). Previous studies suggest that most circRNAs are preferentially expressed in cytoplasm versus nucleus [10, 22]. Consistent with this finding, we reported that circSCAP was mainly distributed in cytoplasm of NSCLC cells (Fig. 1F and G). Generally, circRNAs located in cytoplasm likely function through sponging miRNAs [19, 20]. However, we unexpectedly found that circSCAP could sponge miR-365b-3p on AGO2 but not exerted tumor-suppressing function through it. For lung cancer, many studies have discussed the role of circRNAs as miRNA sponges [11, 30], but little has been documented about circRNAs functioning through binding proteins. In the present study, we report that circSCAP mainly functions through binding to SF3A3 protein, which extends our knowledge of how circRNAs play roles in NSCLC.

SF3A3 is a core U2 snRNP component that is selectively translated by a specialized RNA regulon and exerts the effect through regulating RNA splicing [33]. Despite splicing event of RNA is indispensable for the tumorigenesis [34, 35], the specific role and underlying mechanism is not well understood. A previous report illustrated that HeLa cells viability was decreased tremendously after transfection with siRNA targeting SF3a subunits [36]. Depletion of SF3A3 does not favor the survival of human primary fibroblasts, and results in lower proliferation of breast cancer cells [33]. Also, targeting splice factors  are reported to repress NSCLC effectively [25], but the detailed mechanism remains elusive. Since circSCAP directly bound to SF3A3, we also studied the clinical significance and function of dysregulated SF3A3 in NSCLC. We reported that SF3A3 was highly expressed and higher SF3A3 level was associated with a worse prognosis in lung cancer. Consistent with previous studies, we observed that silencing SF3A3 could suppress proliferation and colony formation ability of NSCLC cells (Figure S6J-L). Additionally, we demonstrated that SF3A3 overexpression obviously facilitated tumor growth in vivo (Fig. 7C and Figure S8A). Collectively, our data confirmed SF3A3 was an oncogenic splicing factor in NSCLC.

The dysfunction of RNA alternative splicing (AS) are detectable in many human cancers [37]. For example, Bcl-x generates two alternatively spliced isoforms, the short isoform (Bcl-xS) and the long isoform (Bcl-xL), among which Bcl-xL has been reported to influence the apoptosis of hepatocellular carcinoma cells [38]. In NSCLC, it has been documented that silencing of SF3A3 could affect the alternative splicing of MDM4 and enhance the generation of MDM4-S [25]. MDM4-S is produced as a result of MDM4 exon 6 skipping, and plays critical roles in inhibiting proliferation of melanoma [39]. In this study, circSCAP was found to promote the decrease of SF3A3 and we confirmed SF3A3 decrease could enhance the alternative splicing process of MDM4 to MDM4-S, leading to the increase of MDM4-S transcript level. Moreover, MDM4-S is reported to be a strong p53 activator [40]. Therefore, we postulate the circSCAP-regulated decrease of SF3A3 might suppress the malignance of NSCLC cells via MDM4-S-driven p53 signaling activation. Expectedly, we observed that SF3A3 knock-down could activate p53 signaling and repress NSCLC cells malignance, while p53 inhibitor could rescued this phenotype. This finding suggests that SF3A3 decrease exerts tumor-suppressing function mainly through MDM4-S-mediated p53 signaling activation. As we have well known, wild-type p53 signaling activation could suppress the cell cycle of cancer cells. We reported here circSCAP-regulated SF3A3 decrease could block the cell cycle transition from G1 to S phase and thus inhibit proliferation of NSCLC cells by activating p53 signaling. In lung cancer, approximately 60% of patients harbor p53 mutations and 40% of patients are with wild-type p53 [41]. p53 mutations mainly play oncogenic roles but wild-type p53 plays tumor-suppressing roles in lung cancer. Here, we found that circSCAP functioned through circSCAP/SF3A3/p53 axis implying that circSCAP might play a tumor-suppressing role in NSCLC cells with wild-type p53. Of note, circSCAP was also downregulated in NSCLC cell lines with p53 mutation (Fig. 2A, HCC-827, PC9 and H1299). As we reported in the study that in NSCLC cells harboring wild-type p53 (A549 and H1650), circSCAP inhibited the malignance of NSCLC cells through SF3A3/p53 signaling. And circSCAP was downregulated in both p53 wild type or mutant cells. To explore whether circSCAP functioned the same way in NSCLC cells with p53 mutation, we firstly detected the background level of p53 in all five NSCLC cell lines used in this study including two cell lines with wild-type p53 (A549 and H1650), two another cell lines with inactivating p53 mutation (PC9 and HCC827) and one cell line with p53 null mutation (H1299). We found that cell lines harboring wild-type p53 (A549 and H1650) exhibited higher p53 signaling when compared to mutant p53 cell lines. The cell lines harboring inactivating p53 mutation (PC9 and HCC827) also displayed a small degree of activated p53 signaling when compared to p53-null cell line H1299 (Figure S10A), implying that some lung cancer cells with p53 mutation retained partial p53 activity. Next, we performed CCK8 and transwell assay to explore the role of circSCAP in NSCLC cell lines with p53 mutation, and the results showed that in the mutant cell lines with partial activated p53 signaling circSCAP exerted a less tumor-suppressing function (Figure S10B,C,F-I) when compared to cell lines with wild-type p53 (Figs. 2 and 3), but no function in cell line with p53 null mutation (Figure S10D-I). And circSCAP also functioned through SF3A3/p53 signaling in mutant cells with partial p53 activation (Figure S11A-F) but no tumor-suppressing function in H1299 with p53 null mutation. Therefore, circSCAP exerts a small degree of tumor-suppressing roles in p53 mutation NSCLC cells with partial p53 activation but no function in p53 null mutation NSCLC cells. As to the role of the downregulation of circSCAP in p53 null mutant NSCLC cells, much more investigations will be needed in the future.

## Conclusions

**Fig. 9:**
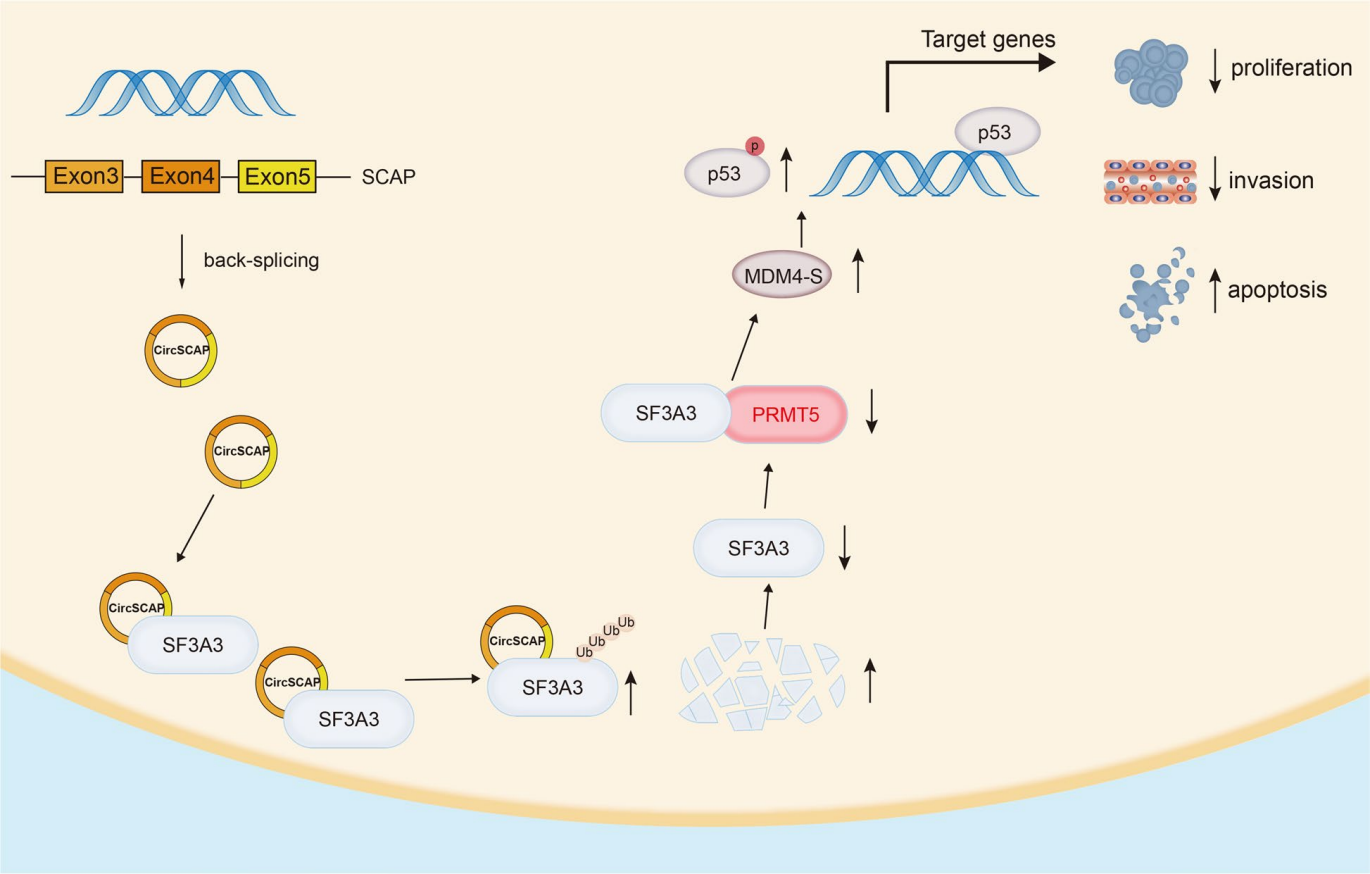
A working model of circSCAP on inhibiting progression of lung cancer via downregulating SF3A3 protein level and activating p53 signaling

In summary, circSCAP directly binds to SF3A3 and promotes its degradation, resulting in the elevated MDM4-S level that activates p53 signaling and subsequently inhibits the malignance of NSCLC (Fig. 9). Our findings provide a novel and potent biomarker for prognosis predication and a circSCAP/SF3A3/p53 regulating axis for future targeting therapy in NSCLC.

## Supplementary Information

**Additional file 1: Figure S1. **CircSCAP is significantly down-regulated in lung cancer.

**Additional file 2: Figure S2.** CircSCAP inhibits the migration but promotes the apoptosis of lung cancer cells in vitro.

**Additional file 3: Figure S3.** CircSCAP functions in lung cancer cells not through sponging microRNAs.

**Additional file 4: Figure S4.** CircSCAP suppresses spliceosome pathway but enhances ubiquitin protein protease pathway.

**Additional file 5: Figure S5.** CircSCAP weakens SF3A3 protein by enhancing its ubiquitination.

**Additional file 6: Figure S6.** SF3A3 acts as an oncogene in lung cancer.

**Additional file 7: Figure S7.** SF3A3 overexpression restores the impaired colony formation ability of lung cancer cells caused by circSCAP.

**Additional file 8: Figure S8.** SF3A3 overexpression rescues the phenotype of lung cancer cells caused by circSCAP in vivo.

**Additional file 9: Figure S9.** SF3A3 decrease suppresses NSCLC malignance by impairing SF3A3/PRMT5 complex-mediated p53 signaling activation.

**Additional file 10: Figure S10.** Background level of p53 signaling determines the role of circSCAP.

**Additional file 11: Figure S11.** CircSCAP functions through SF3A3/p53 axis in NSCLC cell lines with p53 activation but not in cells with p53 null.

**Additional file 12: Table S1.** The sequences of oligonucleotides used for transfection.

**Additional file 13: Table S2.** Primer sequences used in RT-qPCR and PCR analysis.

**Additional file 14: Table S3.** The probe sequences used in this study.

**Additional file 15: Worksheet.** The list of differentially identified proteins by the Mass spectrometry (MS) assay.

## Data Availability

All data in our study are available upon request.

## Abbreviations

CircRNAs: Circular RNAs
NSCLC: Non-small cell lung cancer
SF3A3: Splicing factor 3a subunit 3
PRMT5: Protein arginine methyltransferase 5
miRNA: MicroRNA
siRNA: Small interfering RNA
NC: Negative control
PBS: Phosphate-buffered saline
PMSF: Phenylmethylsulphonyl fluoride
GAPDH: Glyceraldehyde 3-phosphate dehydrogenase
DEcircRNAs: Differentially expressed circRNAs
RIP: RNA immunoprecipitation
FISH: Fluorescence in situ hybridization
IP: Immunoprecipitation
ROC: Receiver operating characteristic
H&E: Hematoxylin and eosin
IHC: Immunohistochemistry
LncRNA: Long non-coding RNAs
ceRNAs: Endogenous RNAs
MS: Mass spectrometry
CHX: Cycloheximide
CO-IP: Coimmunoprecipitation
GSEA: Gene sets enrichment analysis
AS: Alternative splicing
