# Supplementary figures and images for: CircSCAP interacts with SF3A3 to inhibit the malignance of non-small cell lung cancer by activating p53 signaling
Source: J Exp Clin Cancer Res. 2022 Apr 1;41:120. doi: 10.1186/s13046-022-02299-0 (PMC8973551; doi:10.1186/s13046-022-02299-0)

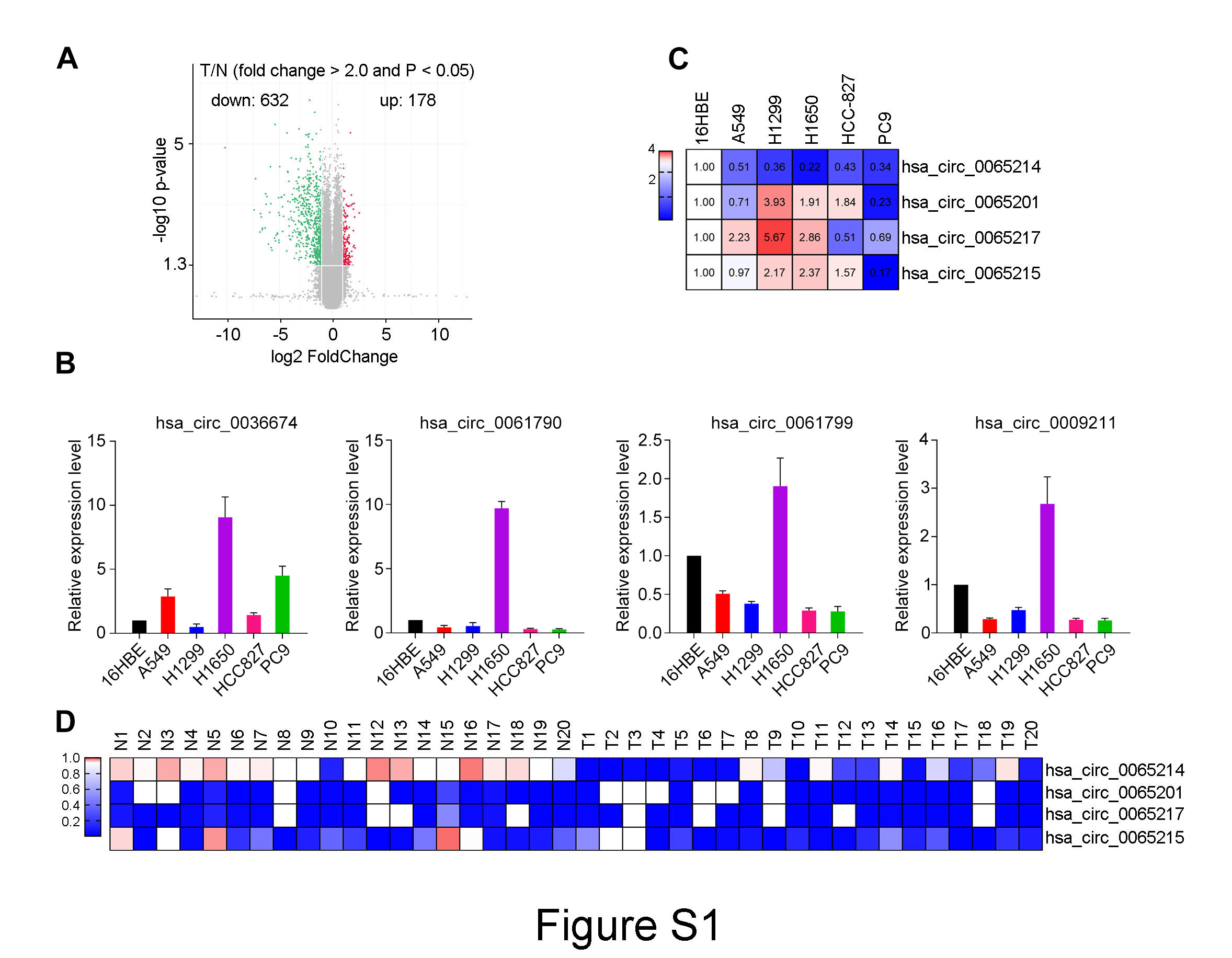

Supplement: Supplementary file 1 — Additional file 1: Figure S1. CircSCAP is significantly down-regulated in lung cancer. [file 13046_2022_2299_MOESM1_ESM.jpg]

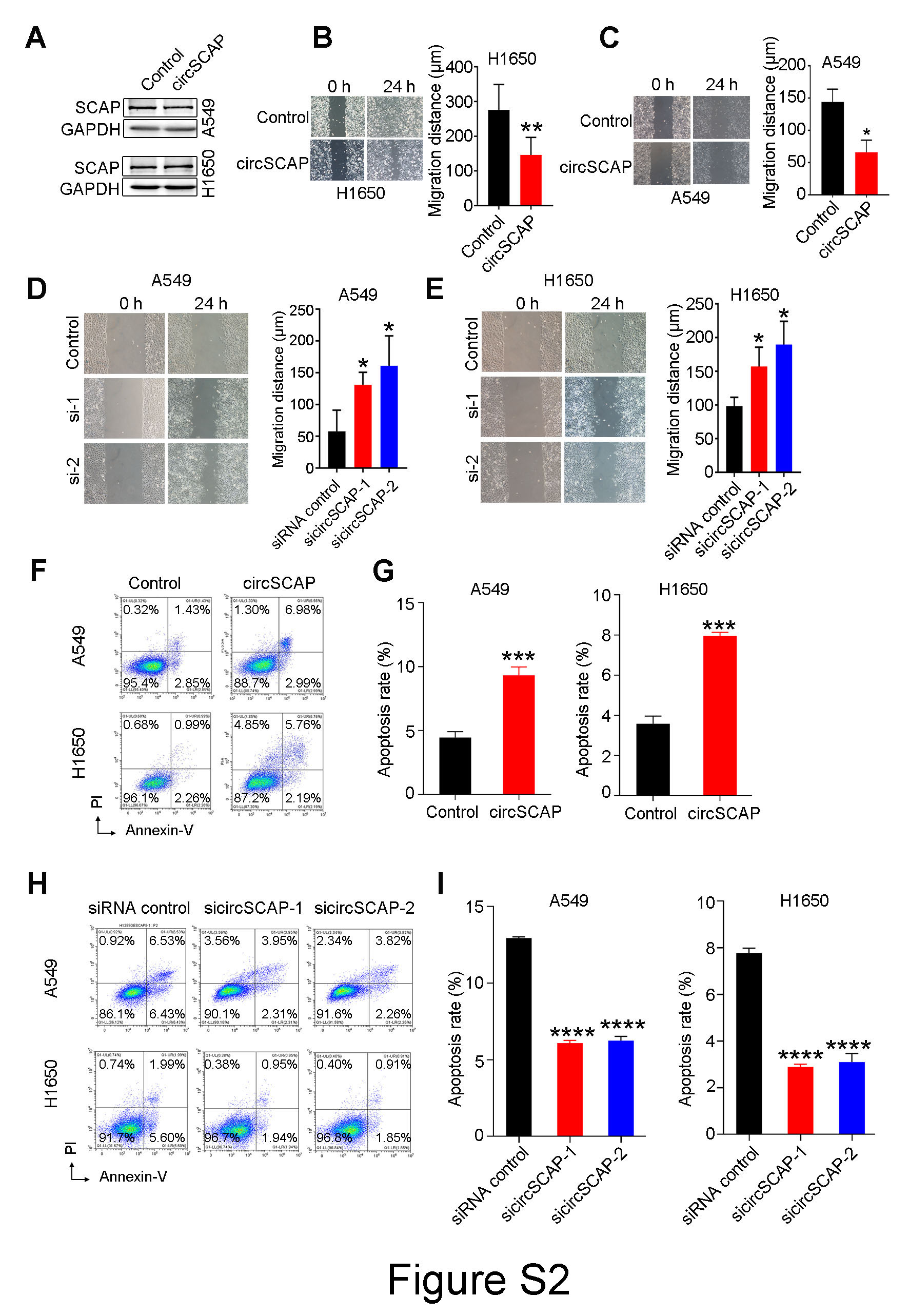

Supplement: Supplementary file 2 — Additional file 2: Figure S2. CircSCAP inhibits the migration but promotes the apoptosis of lung cancer cells in vitro. [file 13046_2022_2299_MOESM2_ESM.jpg]

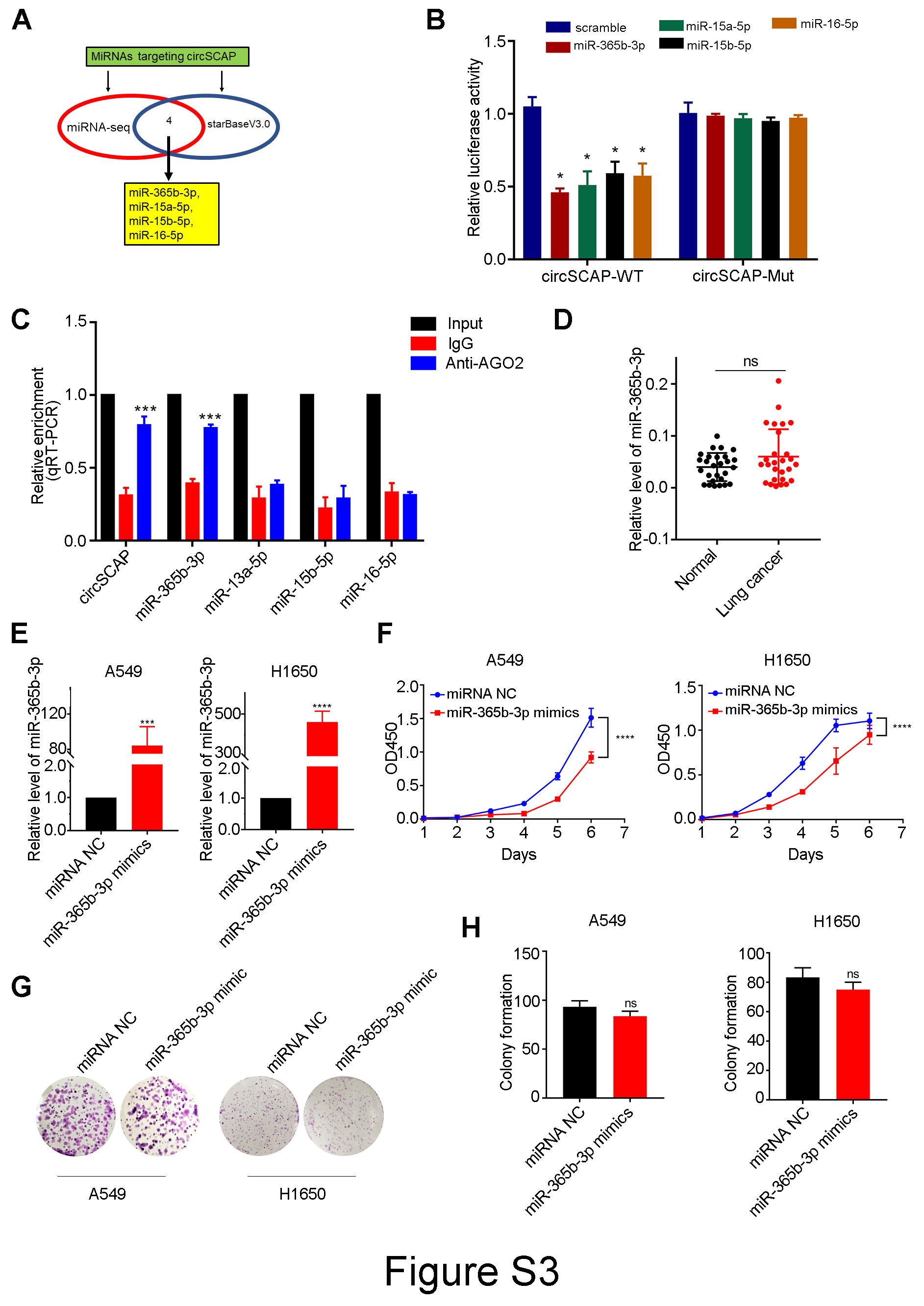

Supplement: Supplementary file 3 — Additional file 3: Figure S3. CircSCAP functions in lung cancer cells not through sponging microRNAs. [file 13046_2022_2299_MOESM3_ESM.jpg]

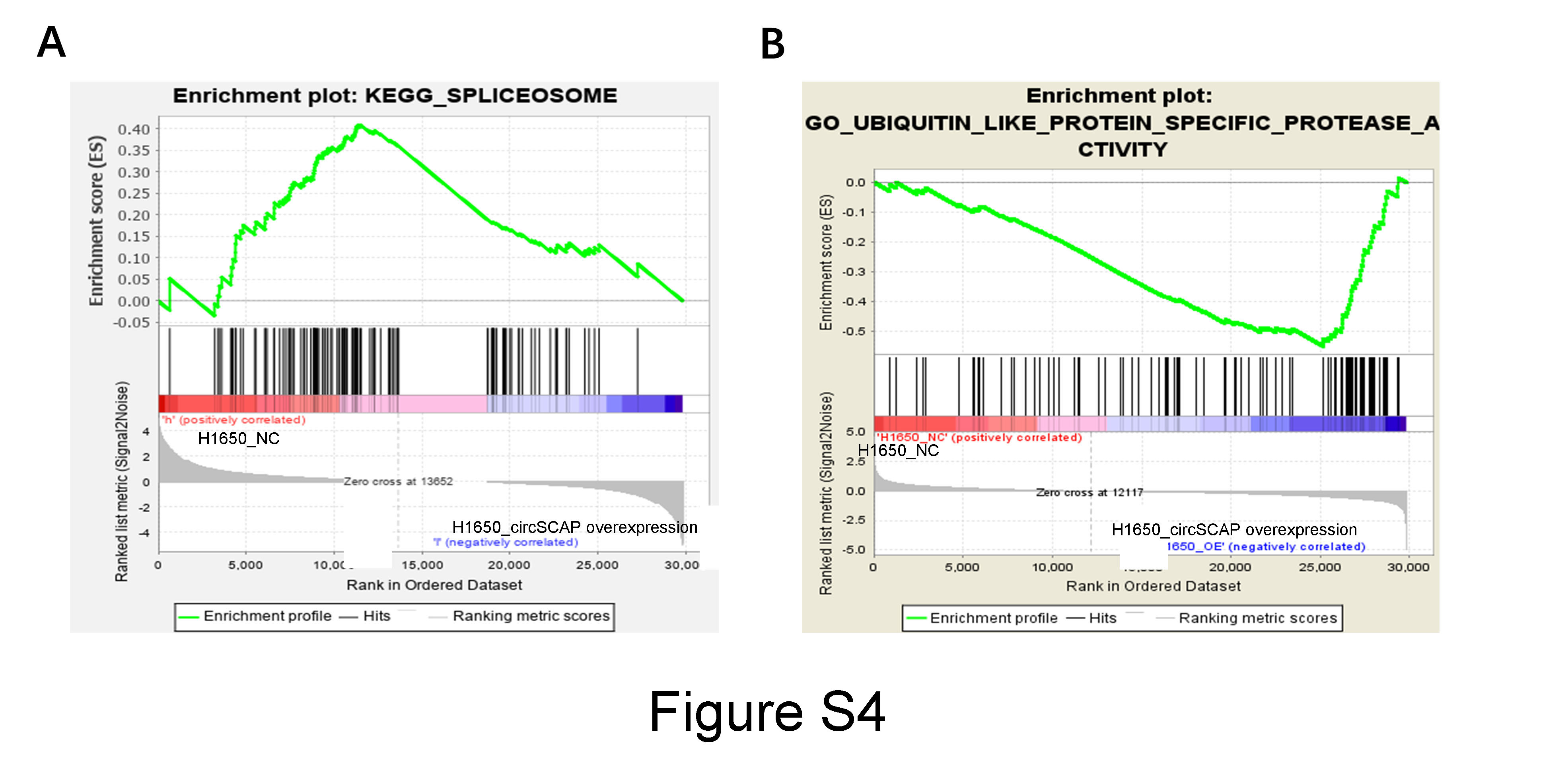

Supplement: Supplementary file 4 — Additional file 4: Figure S4. CircSCAP suppresses spliceosome pathway but enhances ubiquitin protein protease pathway. [file 13046_2022_2299_MOESM4_ESM.jpg]

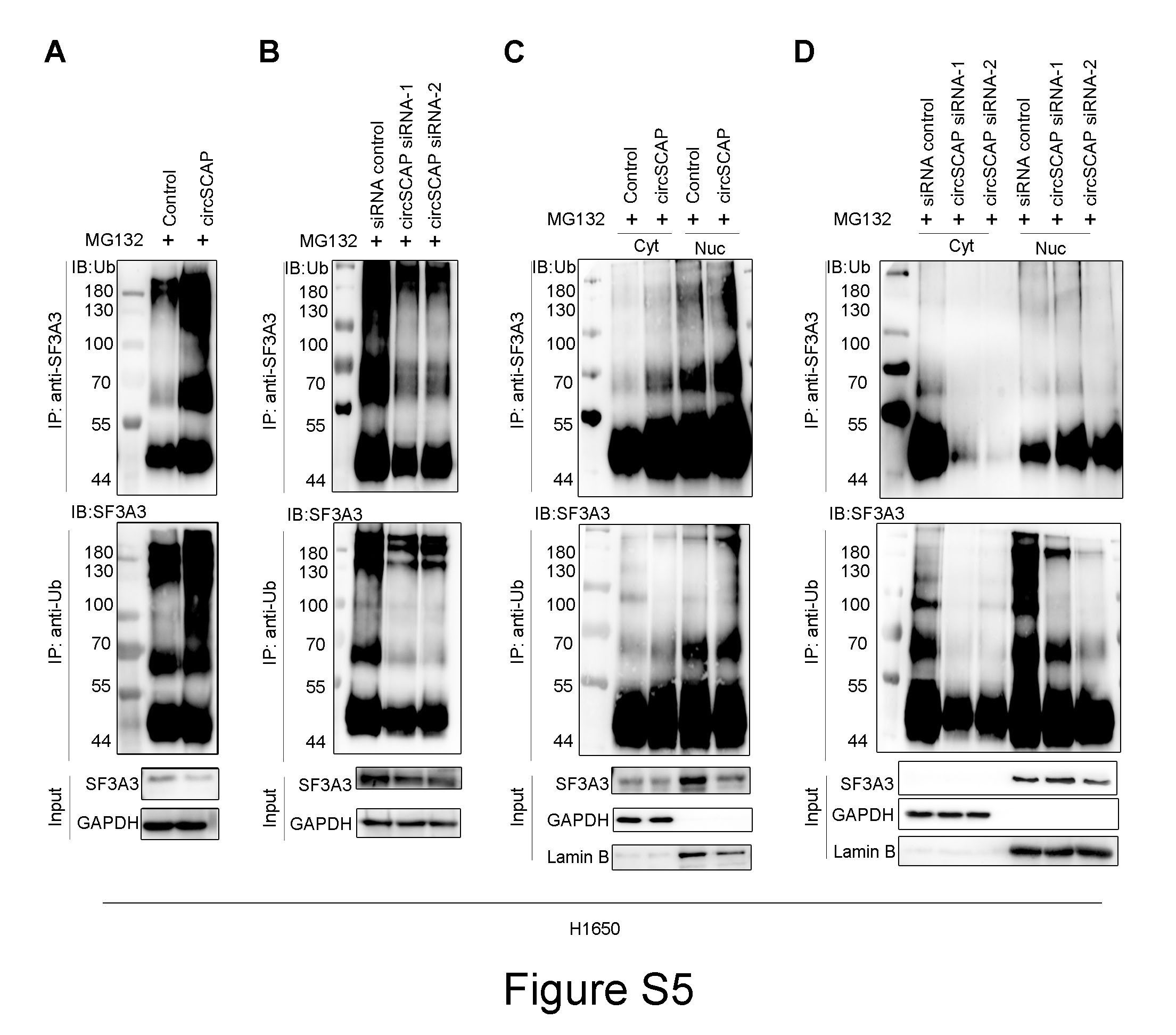

Supplement: Supplementary file 5 — Additional file 5: Figure S5. CircSCAP weakens SF3A3 protein by enhancing its ubiquitination. [file 13046_2022_2299_MOESM5_ESM.jpg]

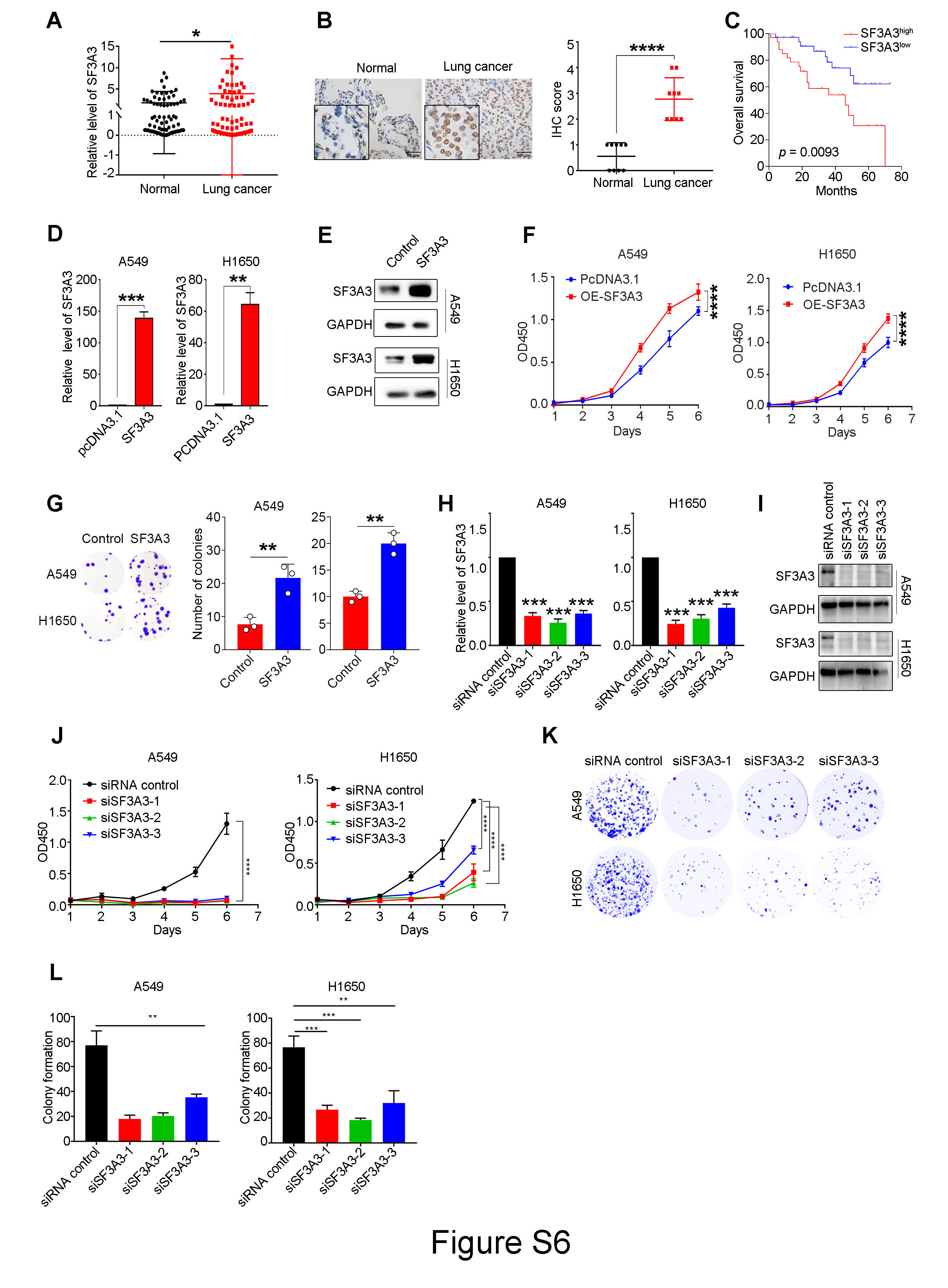

Supplement: Supplementary file 6 — Additional file 6: Figure S6. SF3A3 acts as an oncogene in lung cancer. [file 13046_2022_2299_MOESM6_ESM.jpg]

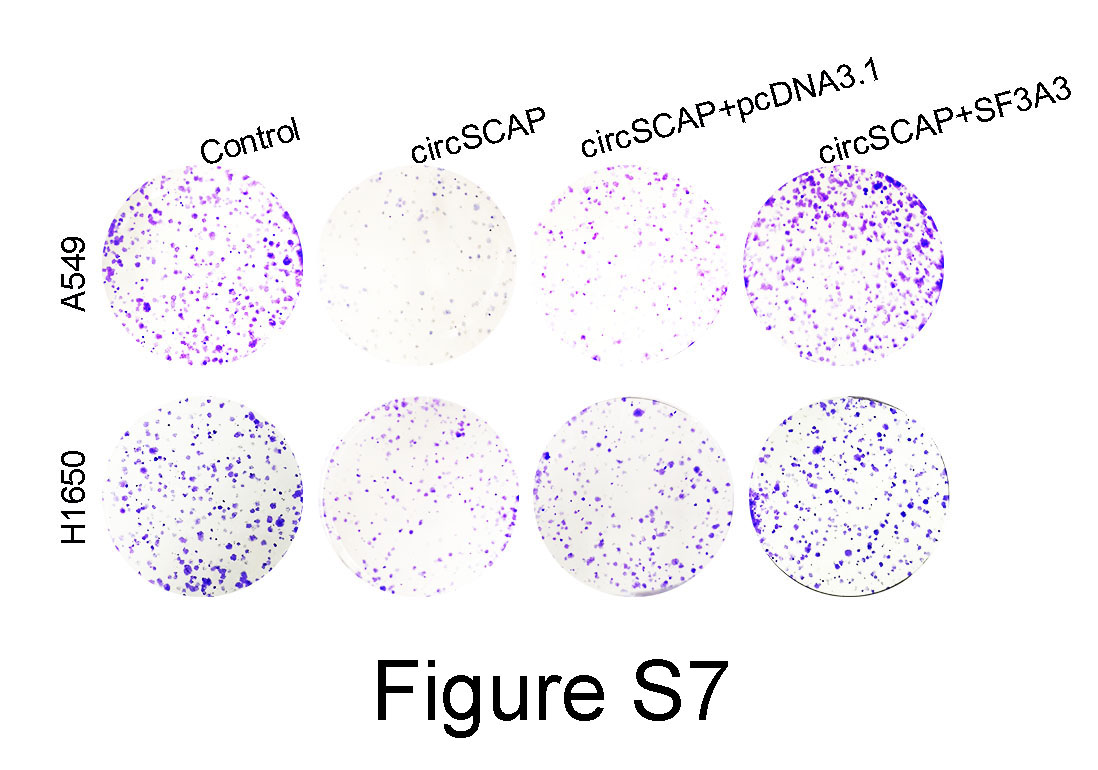

Supplement: Supplementary file 7 — Additional file 7: Figure S7. SF3A3 overexpression restores the impaired colony formation ability of lung cancer cells caused by circSCAP. [file 13046_2022_2299_MOESM7_ESM.jpg]

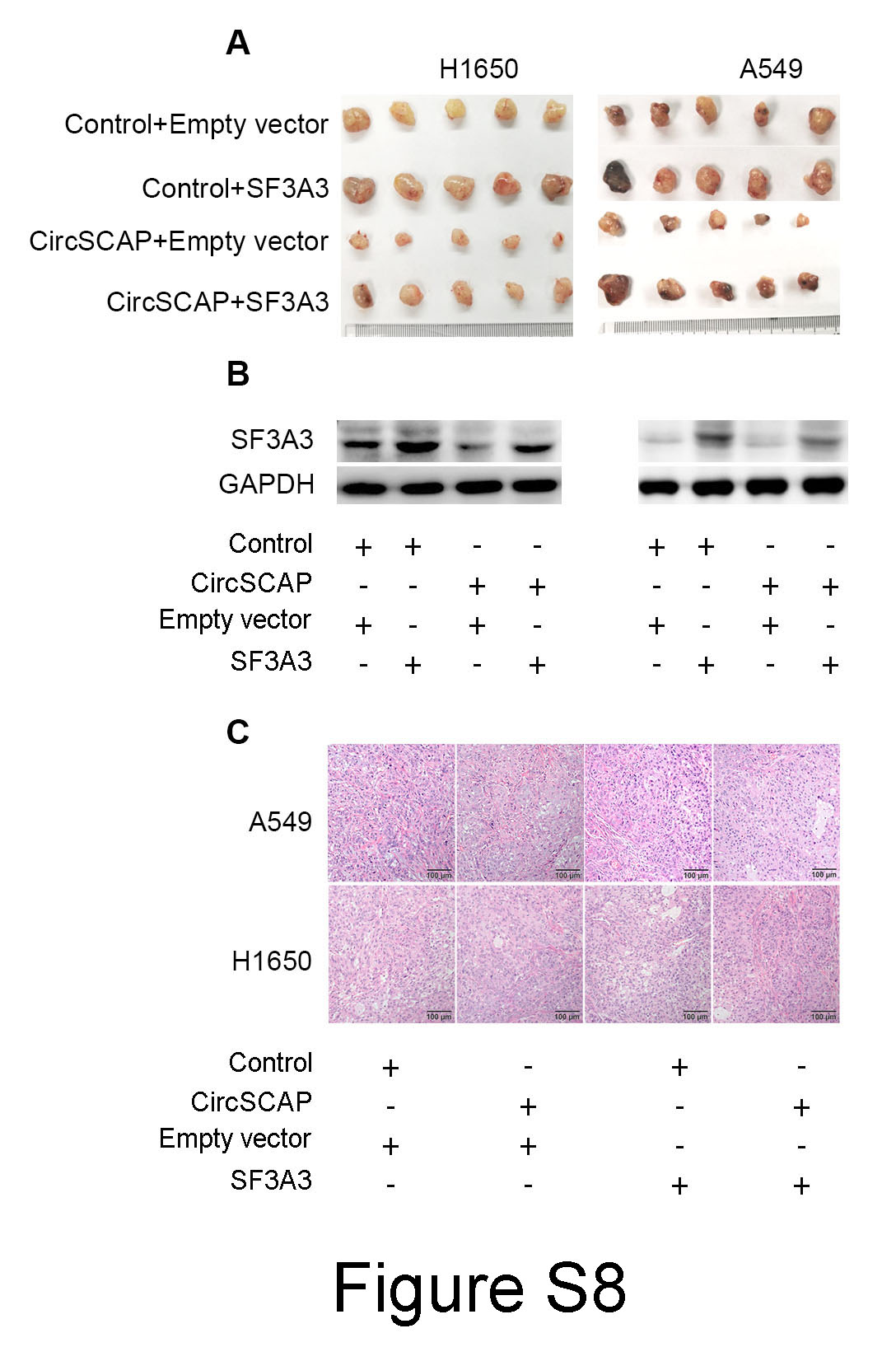

Supplement: Supplementary file 8 — Additional file 8: Figure S8. SF3A3 overexpression rescues the phenotype of lung cancer cells caused by circSCAP in vivo. [file 13046_2022_2299_MOESM8_ESM.jpg]

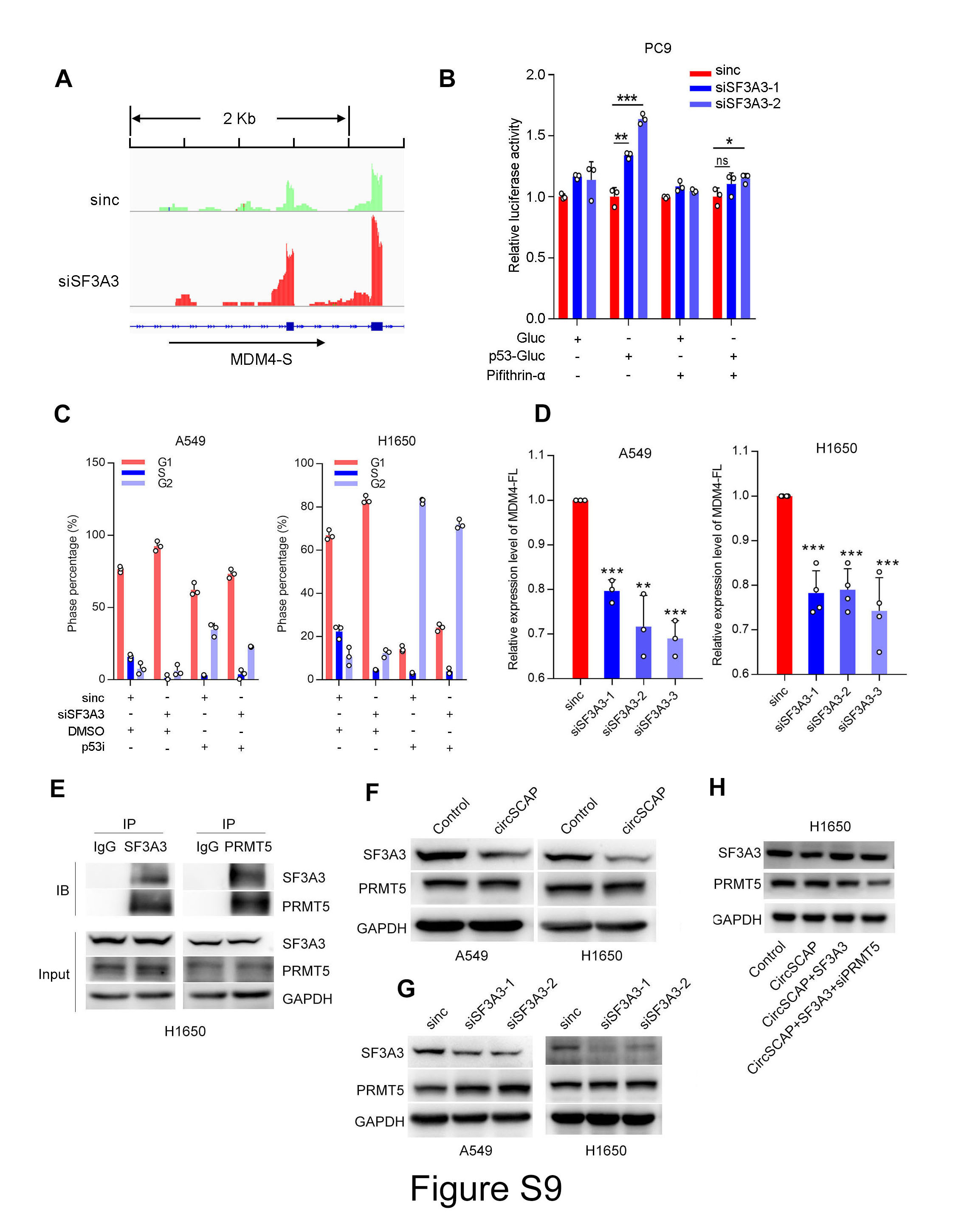

Supplement: Supplementary file 9 — Additional file 9: Figure S9. SF3A3 decrease suppresses NSCLC malignance by impairing SF3A3/PRMT5 complex-mediated p53 signaling activation. [file 13046_2022_2299_MOESM9_ESM.jpg]

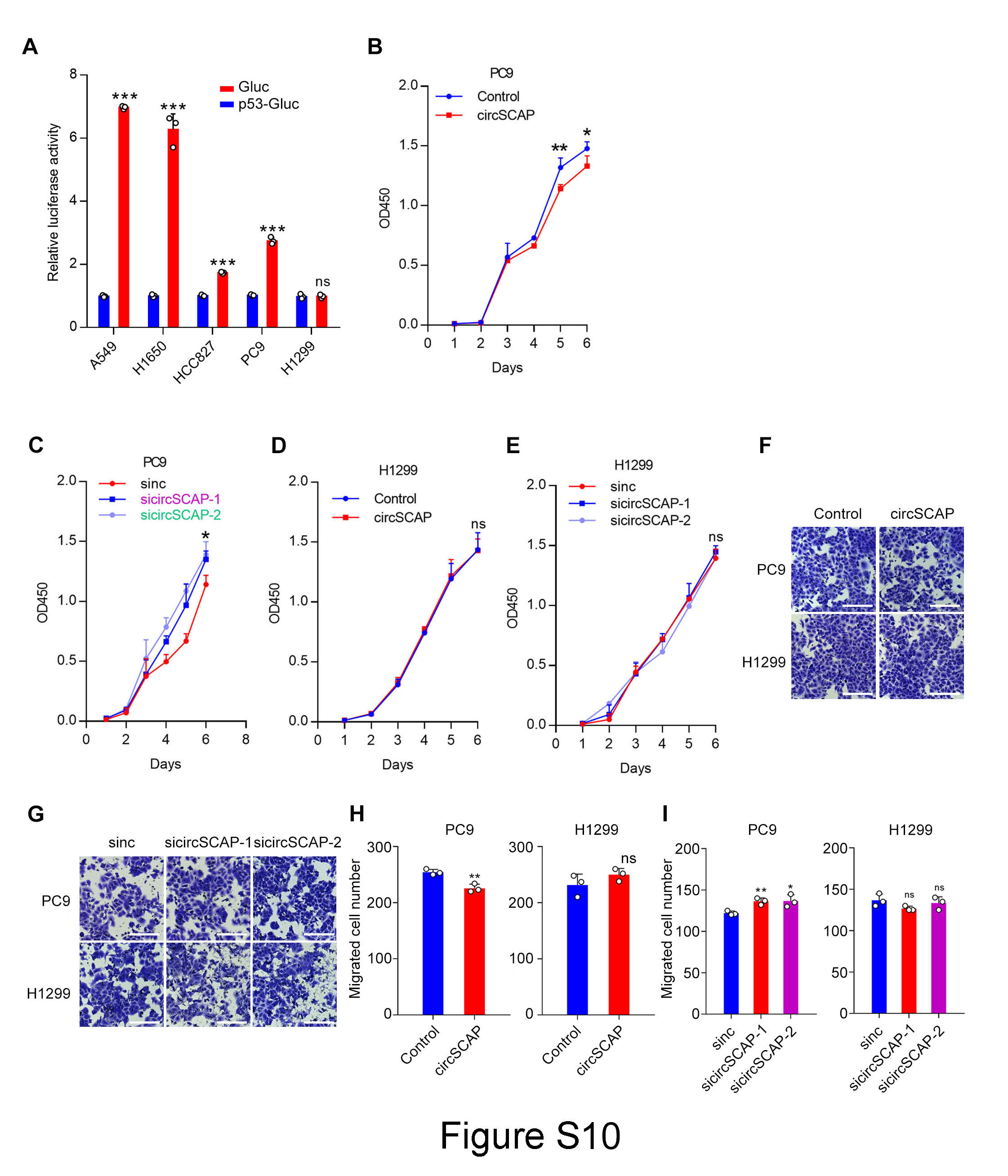

Supplement: Supplementary file 10 — Additional file 10: Figure S10. Background level of p53 signaling determines the role of circSCAP. [file 13046_2022_2299_MOESM10_ESM.jpg]

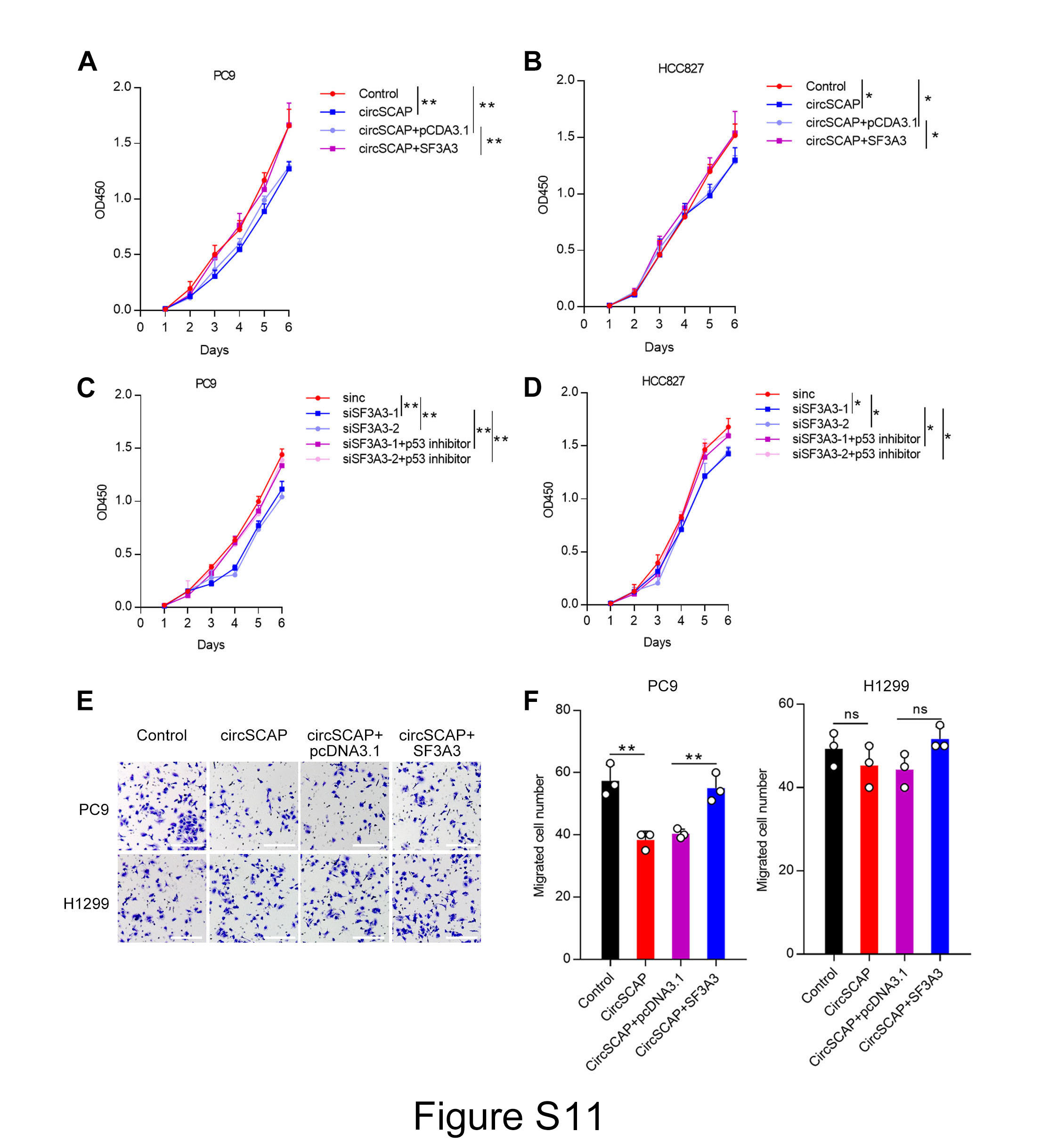

Supplement: Supplementary file 11 — Additional file 11: Figure S11. CircSCAP functions through SF3A3/p53 axis in NSCLC cell lines with p53 activation but not in cells with p53 null. [file 13046_2022_2299_MOESM11_ESM.jpg]
