# Supplementary material for: CircSCAP interacts with SF3A3 to inhibit the malignance of non-small cell lung cancer by activating p53 signaling
Source: J Exp Clin Cancer Res. 2022 Apr 1;41:120. doi: 10.1186/s13046-022-02299-0 (PMC8973551; doi:10.1186/s13046-022-02299-0)
Supplement: Supplementary file 12 — Additional file 12: Table S1. The sequences of oligonucleotides used for transfection. [file 13046_2022_2299_MOESM12_ESM.docx]

**Table S1: The sequences of oligonucleotides used for transfection.**

| **Name** | **Sense 5’-3’** | **Anti-sense 5’-3’** |
| --- | --- | --- |
| hsa_circ_0065214 siRNA 1 | CCUCAAUGGCGGCUACCCATT | UGGGUAGCCGCCAUUGAGGTT |
| hsa_circ_0065214 siRNA 2 | GGCGGCUACCCACUGCUGATT | UCAGCAGUGGGUAGCCGCCTT |
| SF3A3 si-1 | CCUGAAGGCUCGAGAGAAUTT | AUUCUCUCGAGCCUUCAGGTT |
| SF3A3 si-2 | GGAAGAUUCAGGCUGAGUUTT | AACUCAGCCUGAAUCUUCCTT |
| SF3A3 si-3 | GCCCAGAGACUAUUCAGUATT | UACUGAAUAGUCUCUGGGCTT |
| PRMT5 siRNA | GGGACUGGAAUACGCUAAUTT | AUUAGCGUAUUCCAGUCCCTT |
| NC | UUCUCCGAACGUGUCACGUdTdT | ACGUGACACGUUCGGAGAAdTdT |
| MiR-365b-3p mimics | UAAUGCCCCUAAAAAUCCUUAU | AUAAGGAUUUUUAGGGGCAUUA |
| MiR-365b-3p inhibitor | - | AUAAGGAUUUUUAGGGGCAUUA |
| MiR-15a-5p mimics | UAGCAGCACAUAAUGGUUUGUG | CACAAACCAUUAUGUGCUGCUA |
| MiR-15b-5p mimics | UAGCAGCACAUCAUGGUUUACA | UGUAAACCAUGAUGUGCUGCUA |
| MiR-16-5p mimics | UAGCAGCACGUAAAUAUUGGCG | CGCCAAUAUUUACGUGCUGCUA |
| Mimics NC | UUUGUACUACACAAAAGUACUG | CAGUACUUUUGUGUAGUACAAA |
| Inhibitor NC | - | CAGUACUUUUGUGUAGUACAAA |
