# Supplementary material for: CircSCAP interacts with SF3A3 to inhibit the malignance of non-small cell lung cancer by activating p53 signaling
Source: J Exp Clin Cancer Res. 2022 Apr 1;41:120. doi: 10.1186/s13046-022-02299-0 (PMC8973551; doi:10.1186/s13046-022-02299-0)
Supplement: Supplementary file 13 — Additional file 13: Table S2. Primer sequences used in RT-qPCR and PCR analysis. [file 13046_2022_2299_MOESM13_ESM.docx]

**Table S2: Primer sequences used in RT-qPCR and PCR analysis**

| **Primers for q-PCR** | **Sequences** |
| --- | --- |
| GAPDH F | TTGGCCAGGGGTGCTAAG |
| GAPDH R | AGCCAAAAGGGTCATCATCTC |
| hsa_circ_0065214 F | GGGACTCTGCACACTCTTCG |
| hsa_circ_0065214 R | GGCACCCACATACCACTCAG |
| hsa_circ_0065215 F | GGGACTCTGCACACTCTTCG |
| hsa_circ_0065215 R | CCTCAGCCGAAGTCACCTTG |
| hsa_circ_0065201 F | AGTGCCGAGGGTTCCATCT |
| hsa_circ_0065201 R | CTGGGGTGCAGAAGCATCA |
| hsa_circ_0065217 F | TTTGGTGTTCCTGGGAAGTACAG |
| hsa_circ_0065217 R | ACAAATATCTGCTGGACATAAGCC |
| Divergent hsa_circ_0065214 F | TTGTGTCTGCAAGTGACCGA |
| Divergent hsa_circ_0065214 R | CAGGGTTTTAGGCTCGTGCT |
| hsa_circ_0036674 F | TAGAGGCTTTCGGCTTTTTG |
| hsa_circ_0036674 R | ATCTTCCACCGAGCTGTGTC |
| hsa_circ_0061799 F | CCCAGTGTTTTAGGAGCATGA |
| hsa_circ_0061799 R | GTGTGATGAGCTCGCTGGTA |
| hsa_circ_0009211 F | ATCAAGGGCCGGAAATAAAG |
| hsa_circ_0009211 R | CTTCAGCTCTGACACCGACA |
| hsa_circ_0061790 F | TTAGGAGCATGAGTGCCGTG |
| hsa_circ_0061790 R | TCATTCTCCAAACCTGCGCT |
| liner SCAP F | TATCTCGGGCCTTCTACAACC |
| liner SCAP R | GGGGCGAGTAATCCTTCACA |
| SF3A3 F | GTCATGGCTAAAGAGATGCTCAC |
| SF3A3 R | TCCTCCTTTCGTAATCCATCCTT |
| PRMT5 F | AGAACCGTCCTCCACCTA |
| PRMT5 R | CTCCCAGCACCATCAGTA |
| mirnauniversal | GAAAGAAGGCGAGGAGCAGATCGAGGAAGAAGACGGAAGAATGTGCGTCTCGCCTTCTTTCNNNNNNNN |
| qpcruniversal | TGGTGTCGTGGAGTCG |
| u6 F | CTCGCTTCGGCAGCACA |
| U6 R | AACGCTTCACGAATTTGCGT |
| hsa-miR-365b-3p | ACACTCCAGCTGGGTAATGCCCCTAAAAAT |
| MDM4-S F | CAGCAGGTGCGCAAGGTGAA |
| MDM4-S R | GCACTTTGCTGTAGTAGCAGTG |
