# Supplementary material for: CircSCAP interacts with SF3A3 to inhibit the malignance of non-small cell lung cancer by activating p53 signaling
Source: J Exp Clin Cancer Res. 2022 Apr 1;41:120. doi: 10.1186/s13046-022-02299-0 (PMC8973551; doi:10.1186/s13046-022-02299-0)
Supplement: Supplementary file 14 — Additional file 14: Table S3. The probe sequences used in this study. [file 13046_2022_2299_MOESM14_ESM.docx]

**Table S3: The probe sequences used in this study.**

**RNA pull down**

**① hsa_circ_0065214 sequence**

CGCCCACCCTCAATGGCGGCTACCCACTGCTGAAACTCCCCTTGCCAGGAACAGGACCTGTGGAATTCACCACCCCTGTGAAGGATTACTCGCCCCCACCTGTGGACTCTGACCGCAAACAAGGAGAGCCTACTGAGCAGCCTGAGTGGTATGTGGGTGCCCCGGTGGCTTATGTCCAGCAGATATTTGTGAAGTCCTCAGTGTTTCCCTGGCACAAGAACCTCCTGGCAGTAGATGTATTTCGTTCACCTTTGTCCCGGGCATTCCAACTGGTGGAGGAGATCCGGAACCACGTGCTGAGAGACAGCTCTGGGATCAGGAGCTTGGAGGAGTTGTGTCTGCAAGTGACCGACCTGCTGCCAGGCCTTAGGAAGCTCAGGAACCTACTCCCTGAGCATGGATGCCTGCTGCTGTCCCCTGGGAACTTCTGGCAGAATGACTGGGAACGCTTCCATGCTGATCCTGACATCATTGGGACCATCCACCAGCACGAGCCTAAAACCCTGCAGACTTCAGCCACACTCAAAGACTTGTTATTTGGTGTTCCTGGGAAGTACAGCGGGGTGAGCCTCTACACCAGGAAGAGGATGGTCTCCTACACCATCACCCTGGTCTTCCAGCACTACCATGCCAAGTTCCTGGGCAGCCTGCGTGCCCGCCTGATGCTTCTGCACCCCAGCCCCAACTGCAGCCTTCGGGCGGAGAGCCTGGTCCACGTGCACTTCAAGGAGGAGATTGGTGTCGCTGAGCTCATCCCCCTTGTGACCACCTACATCATCTTGTTTGCCTACATCTACTTCTCCACGCGGAAGATCGACATGGTCAAGTCCAAGTGGGGGCTGGCCCTGGCTGCCGTGGTCACAGTGCTCAGCTCGCTGCTCATGTCTGTGGGACTCTGCACACTCTTCGGCCTGACGCCCACCCTCAATGGCGG

**② LacZ (negative) sequence**

TGGCCGTCGTTTTACAACGTCGTGACTGGGAAAACCCTGGCGTTACCCAACTTAATCGCCTTGCAGCACATCCCCCTTTCGCCAGCTGGCGTAATAGCGAAGAGGCCCGCACCGATCGCCCTTCCCAACAGTTGCGCAGCCTGAATGGCGAATGGCGCCTGATGCGGTATTTTCTCCTTACGCATCTGTGCGGTATTTCACACCGCATATGGTGCACTCTCAGTACAATCTGCTCTGATGCCGCATAG

**RNA-FISH**

**① hsa_circ_0065214 sequence**

5’- Digoxigenin labeling – GCAGTGGGT AGCCGCCATTGAGG -3’

**② U6**

5’- Digoxigenin labeling – CACGAATTTG CGTGTCATCCTT -3’

**Northern blot**

**① hsa_circ_0065214 sequence**

TAATACGACTCACTATAGGGCCCACCCT CAATGGCGGCTACCCACTGCTGAAAC
